# Specialty choices among UK medical students: certainty, confidence and key influences—a national survey (FAST Study)

**DOI:** 10.1136/bmjopen-2025-103061

**Published:** 2025-08-08

**Authors:** Tomas Ferreira, Alexander M Collins, Arthur Handscomb, Benjamin French, Emily Bolton, Amelia Fortescue, Ella Plumb, Oliver Feng, the FAST Collaborative, Megan Fallows

**Affiliations:** 1 University of Bristol Medical School, Bristol, UK; 2 Queen Elizabeth Hospital, Woolwich, Lewisham and Greenwich NHS Trust, London, UK; 3 St George’s University Hospitals NHS Foundation Trust, London, UK; 4 Department of Mathematical Sciences, University of Bath, Bath, UK

**Keywords:** Medical Education & Training, Health Education, Health Services

## Abstract

**Abstract:**

**Objective:**

To explore factors influencing UK medical students’ specialty choices and examine variations in these influences across demographic groups and stages of training.

**Design:**

National, cross-sectional online survey.

**Setting:**

All 44 UK medical schools recognised by the General Medical Council.

**Participants:**

8,395 medical students.

**Primary and secondary outcomes:**

The primary outcome was the specialty preferences of UK medical students. The secondary outcomes were factors behind these preferences and how these factors vary across demographic groups and different stages of training.

**Results:**

General Practice (15.3%), Paediatrics (10.6%) and Anaesthetics (9.9%) were the most preferred specialties among final-year students. Work-life balance (84.1%), compatibility with family life (78.2%), positive training experiences (85.2%) and future specialty outlook (74.9%) were key factors influencing specialty choice. Only 23.1% of students felt confident about securing a specialty training post, with confidence higher among males (OR 1.36, 95% CI 1.21 to 1.52, p<0.0001) and privately educated students (OR 1.18, CI 1.03 to 1.35, p=0.02). Males were also more certain about their career choices (OR 1.19, 95% CI 1.07 to 1.31, p<0.0001). Confidence in securing a training place was positively associated with extracurricular achievements, including having a PubMed-indexed publication (OR 1.67, 95% CI 1.39 to 2.00, p<0.0001).

**Conclusions:**

This study highlights disparities in specialty preferences and influencing factors among UK medical students. A focus on improving career guidance, exposure to various specialties and supporting equitable access to training opportunities is essential for fostering a motivated and sustainable medical workforce.

**Strengths and limitations of this study**

- This is the largest study of UK medical students’ specialty preferences to date, offering unprecedented insight into the factors influencing career intentions across all years of study.
- The study provides comprehensive subgroup analyses across demographics such as gender, ethnicity and schooling type, delivering a nuanced understanding of disparities in confidence, certainty and influencing factors.
- The national scope and large sample size increase the reliability and generalisability of the findings to the wider UK medical student population.
- Although the cross-sectional design limits the ability to draw causal inference, it offers a valuable snapshot of current trends and priorities among medical students.
- Reliance on self-reported data may introduce some bias, but the large sample size and anonymity likely mitigated these effects, contributing to the reliability of the results.

## Introduction

 In the UK, medical education is undertaken either as a 5-year to 6-year undergraduate degree or a 4-year postgraduate degree.^1 2^ On graduation, doctors receive provisional registration with the General Medical Council (GMC), enabling them to begin training within the UK’s National Health Service (NHS). This training begins with the mandatory UK Foundation Programme (FP), a 2-year scheme consisting of six rotations covering various specialties. Although full GMC registration is achieved after the first year of the FP, the programme’s completion is necessary to pursue specialisation.^1 2^

Following foundation training, doctors enter a competitive selection process to progress into specialty training (residency). Depending on their chosen field, they can apply for either ‘core’ training in a broad discipline, such as medicine, surgery or anaesthetics, or directly into a particular specialty that offers uninterrupted ‘run-through’ training, such as general practice or radiology. Applications are generally assessed based on a combination of academic and clinical achievements that comprise their portfolios, including research publications, audits, teaching experience and additional degrees, often in tandem with performance in examinations and interview.^3 4^

Gaining admission to specialist training programmes is conditional on successful application via these nationally administered processes and has become highly competitive in recent years.^5 6^ Concurrently, over a third of medical students reportedly intend to leave the NHS within 2 years of graduation.^7 8^ There is therefore a critical need to closely monitor prospective doctors’ career intentions and the underlying motivations, thereby informing policy adjustments and educational interventions.

Determining which specialty to pursue as a career is a challenging question faced by medical students worldwide, and there is an interplay of factors which influence this decision. Certain factors may be intrinsic to a given specialty and therefore either serve to attract or repel students depending on their interests, such as perceived work-life balance, prestige or financial reward.^9^,^19^ Moreover, individual or incidental factors, such as students’ demographic backgrounds, their experiences at medical school, influence from family or mentors and personal life experiences or goals, have also been identified as relevant in this process.

The literature encompasses both UK-based and non-UK-based studies investigating the factors affecting doctors’ and medical students’ propensity towards selected specialties and reasons therefor.^20^ However, there is a notable lack of large-scale studies that comprehensively summarise the specialty preferences of UK medical students across all years of study and explore the factors underpinning these decisions.^20^ The Factors Affecting Specialty Training preference (FAST) study addresses this gap by exploring the views of more than 8000 medical students, making it the largest ever survey of UK medical students’ specialty preferences and influencing factors, and the second largest study of UK medical students overall. This extensive dataset provides unprecedented insights into the specialty choices of UK medical students and the demographic and experiential factors that shape these decisions.

This study aimed to explore the factors influencing which specialty pathways medical students wish to pursue. Secondary aims included determining variations in these influences by region, stage of training and across demographic groups. Additionally, readers may find further insights in our sister publication, which examines medical students’ involvement in extracurricular achievements and their role in shaping students’ career trajectories (ref).

## Methods

### Study design

FAST was a national, multicentre, cross-sectional study of UK medical students, conducted in accordance with its protocol,^20^ following a similar methodology to the AIMS study.^7^ Participants’ responses were recorded via an online survey platform. The survey contained 17 questions structured using a combination of Likert scale matrices, multiple-choice options and free-text entry to facilitate the capture of sentiment nuance and improve precision in the data. Data collection took place between 4 December 2023 and 1 March 2024.

The survey consisted of three sections. Section 1 sought background and demographic information, as well as participants’ consent. Section 2 gathered information on certainty and confidence regarding students’ choice of specialty, and knowledge regarding pursuit of their chosen specialty. Section 3 invited participants to rate the importance of various factors influencing their specialty choice and an optional free-text entry for additional factors. The full questionnaire is available in online supplemental material 1.

The survey was hosted on Qualtrics (Provo, Utah, USA), a General Data Protection Regulation (GDPR) compliant online survey platform which supports both mobile and desktop devices. Anonymous responses were collected and stored on the secure online server Qualtrics. Data were extracted and stored in a password-protected Microsoft Excel (Microsoft, California, USA) file accessible only to the central study team.

### Participant recruitment and eligibility

The survey was distributed through multiple channels, including university mailing lists, student society social media pages, conferences, and personal and medical school social media platforms (such as Facebook, Instagram, LinkedIn and X). A national network of approximately 200 FAST Collaborative members was recruited to maximise distribution, representing all UK medical schools. All UK medical students were eligible to apply. Collaborators liaised within their medical schools to obtain permission to distribute the survey through official channels, and advertised it periodically throughout the data collection period.

All current students in all years at GMC-accredited UK medical schools were eligible to participate in the study. Where new schools had received GMC approval, but were yet to admit any students, they were excluded from this present study. All eligible institutions and approved medical programmes are listed in online supplemental material 2. Responses were received from participants across all 44 eligible schools.

### Data processing and storage

Responses were restricted to a single institutional email address to mitigate the risk of data duplication. Any replicated email entries were removed prior to data analysis. Where repeated entries contained distinct responses, the most recent entry was retained. Entries where respondents did not provide valid institutional email addresses were removed prior to data analysis to preserve the integrity of the study.

### Ethical considerations

As per UK NHS Health Research Authority guidance, NHS Research Ethics Committees review exemption was applied. All data are anonymous, and informed consent was obtained prior to participation.

A Participant Information Sheet (online supplemental material 3) was made available to all participants for informed consent. This outlined the investigation’s rationale and purpose, emphasising its voluntary nature and the anonymity and confidentiality of participation. It was made clear on study materials and the first page of the questionnaire that submitting responses provided consent for data usage. The first question of the survey obtained explicit consent with mandatory completion for survey progression. Consent could be withdrawn at any time, during or after completing the questionnaire by contacting the study lead via an email address which was made available to all participants.

Participants received no compensation for completing the questionnaire. However, participants were entered into a prize draw, with one randomly selected individual receiving a cash prize of £250 once data collection had ceased.

### Patients and public involvement

To identify gaps in knowledge and inform the aims of the project, a literature review was conducted, including similar questionnaires and qualitative studies on students’ and doctors’ perspectives. Feedback from senior clinical staff was also obtained. Questions were revised based on comments received, aiming to ensure they were as non-directive and comprehensive as possible, while remaining unencumbering so as to maximise the number of responses for statistical power.

### Missing values

The survey comprised mostly mandatory questions, with only 360 of 8,395 students opting to withhold information (by selecting ‘Prefer not to say’ for ethnicity, gender and/or schooling). These individuals were excluded from the dataset, and a complete case analysis was performed.

### Predictor variables

The selection of demographic and educational background variables was informed by existing literature on factors influencing specialty choice and career progression among medical students. Prior studies have highlighted significant disparities in career confidence, specialty preference and achievement linked to gender, ethnicity, socioeconomic background, year of study and familial medical connections.^20^,^26^ Therefore, our study sought to evaluate these variables systematically to identify their impact on students’ specialty selection and career planning. Categorical covariates in our statistical models were derived from students’ demographic and educational background responses (table 1). Gender was recorded as a separate variable, while ethnicity was classified into four broad groups rather than specific subgroups. These classifications were chosen to reflect major demographic trends in UK medical education. Since the data were collected directly from students rather than institutions, the closest available proxy for socioeconomic status was school type (ie, state comprehensive, grammar or private) rather than more detailed measures such as family income or Indices of Deprivation.^27^ Additionally, students were asked whether they had a parent or sibling in medicine, given the potential influence of familial guidance, career insights and access to work experience opportunities.

**Table 1:** Demographic characteristics of participants

Characteristics	Number	%
Ethnicity		
Asian or Asian British	2532	30.2%
Black, Black British, Caribbean or African	491	5.9%
Mixed or multiple ethnic groups	447	5.3%
White	4474	53.3%
Other	354	4.2%
Prefer not to say	97	1.2%
Gender		
Female	5727	68.2%
Male	2537	30.2%
Non-binary	78	0.9%
Prefer not to say	53	0.6%
Level of education		
Postgraduate	1505	17.9%
Undergraduate	6890	82.1%
Previous schooling		
Comprehensive state school	4061	48.4%
Selective state school or grammar school	1883	22.4%
Private school (fee-paying)	2180	26.0%
Prefer not to say	271	3.2%
Parent or sibling in Medicine		
Yes	1717	20.5%
No	6678	79.6%
Fee status		
Home	7305	87.0%
EU/EEA	293	3.5%
International (non-EU)	797	9.5%
Year of study		
Year 1	1258	15.0%
Year 2	1613	19.2%
Year 3 (but not penultimate year)	1688	20.1%
Year 4 (but not penultimate or final year)	756	9.0%
Penultimate year	1759	21.0%
Final year	1321	15.7%
Age		
Median (range)	22 (17–51)	

EU/EEA, European Union/European Economic Area.

To account for the variability in UK medical degree durations (ranging from 4 to 6 years), students reported their year of study as Year 1, Year 2, Year 3 (not penultimate), Year 4 (not penultimate or final), Penultimate or Final. Students were also asked whether they had completed an intercalated or previous degree. Furthermore, information was collected on whether students had completed various extracurricular achievements, such as authorship of PubMed-indexed articles, delivery of oral or poster presentations, and participation in audits or leadership roles. These criteria were included as they are explicitly featured in specialty training shortlisting matrices, where applicants are awarded points based on their achievements.^28^ As such, these activities may be directly correlated with a student’s likelihood of securing a competitive specialty training post. Among these, PubMed-indexed authorship was chosen as the primary metric for inclusion in our models, given its high level of commitment and recognition within medical academia.

### Outcome variables

Section 2 of the survey assessed students’ confidence, certainty and perceived knowledge regarding specialty training applications, using a 5-point Likert scale. Section 3 examined the importance of various factors in specialty selection, again rated on a 5-point scale. These factors encompassed work and training characteristics, financial considerations, career security, personal and lifestyle preferences, intellectual and professional growth, perceived prestige, patient and clinical interactions, and workplace diversity. A full list of these variables is available in table 2.

**Table 2:** Influence of various factors on specialty training preferences among final-year medical students

Factors affecting specialty training preference	Influential	Neutral	Not influential
Work and training characteristics			
Work-life balance	84.1%	9.4%	6.5%
Length of specialty training	47.8%	26.9%	25.4%
Level of stress and pressure at work	67.5%	21.1%	11.4%
Level of competition for entry into the specialty	53.3%	26.8%	19.9%
Training structure (run through ie, entry at ST1 vs uncoupled, ie, entry via core training and having to reapply at ST3)	47.6%	28.9%	23.5%
Financial considerations			
Financial remuneration	68.4%	19.9%	11.7%
Potential for private practice earnings	42.8%	23.2%	34.1%
Number of exams and overall cost of specialty training	36.8%	30.5%	32.7%
Career security			
Future outlook of the specialty	74.9%	16.3%	8.9%
Patient and clinical interactions			
Continuity of care with patients	47.1%	27.2%	25.7%
Level of patient interaction	77.7%	15.1%	7.2%
Diversity of patient interactions	67.5%	19.7%	12.9%
Personal and lifestyle considerations			
Compatibility with family life	78.2%	13.3%	8.5%
Out-of-hours demands (OOH shifts)	67.2%	20.8%	12.0%
Preferences regarding working location (eg, tertiary hospital vs district general hospital vs community)	63.7%	21.0%	15.3%
Social and professional perception			
Perceived prestige of specialty	19.8%	22.6%	57.7%
Stereotypes surrounding specialty	17.2%	22.5%	60.3%
Gender split in specialty			
Gender distribution of doctors in the specialty	23.3%	27.2%	49.5%
Demographic preferences			
Preference for working with specific gender groups	13.1%	19.8%	67.2%
Preference for working with specific age groups (eg, geriatrics, paediatrics)	37.9%	20.6%	41.6%
Intellectual and professional growth			
Intellectual challenge	70.6%	18.6%	10.8%
Research opportunities within specialty	36.0%	23.2%	40.9%
Use of advanced technology in the specialty	27.9%	25.4%	46.8%
Use of clinical diagnostic skills vs investigations	53.3%	28.1%	18.6%
Interest in specific conditions	55.1%	24.2%	20.7%
Previous experiences			
Personal experiences of disease	24.2%	20.4%	55.4%
Preclinical positive experiences with the specialty (eg, lectures, tutorials)	49.9%	17.3%	32.9%
Past positive interactions with the specialty (eg, rotations, clinical attachments)	85.2%	8.8%	6.1%
Influence of mentors or role models	63.2%	19.3%	17.5%

Note: ‘Influential’ includes responses marked as ‘Fairly influential’ or ‘Very influential’; ‘Not influential’ includes ‘Not at all influential’ and ‘Fairly uninfluential.’

### Data analysis

Data storage and preprocessing were conducted using Microsoft Excel V.16.71 (Arlington, Virginia, USA), while GraphPad Prism V.10.3.0 (San Diego, California, USA) was used to generate graphs and frequency tables, with percentages rounded to one decimal place. Statistical analyses were performed in RStudio V.1.4.1564 (Boston, Massachusetts, USA) running R V.4.4.1 (Vienna, Austria).

Survey responses were recorded on a 5-point Likert scale. While ordinal logistic regression was initially considered for analysing the relationship between outcome variables and demographic or educational predictors, this method assumes proportional odds, which may not be appropriate for this dataset. Instead, responses were dichotomised by grouping the two highest categories (‘somewhat’ or ‘very’ confident, certain or informed’) and merging the remaining three categories to create a binary outcome variable. Multiple logistic regression models were then fitted, incorporating all relevant predictors outlined in the Methods section.

The model output from the *glm* function was used to obtain ORs for individual covariates, with approximate 95% CIs, using suitable baseline categories. ORs were reported alongside p values to assess statistical significance. Estimates were rounded to two decimal places, and p values below 0.01 were either rounded to two significant figures or denoted as p<0.0001 where appropriate. To account for multiple comparisons, we apply a Bonferroni-adjusted significance threshold of 0.001 (0.05/50), given that fewer than 50 hypothesis tests are conducted in this analysis. However, individual p values remain unadjusted in the results.

### Radar plot generation

To visualise specialty-specific differences in the relative importance of influencing factors, we created radar plots based on composite scores. Students rated 28 individual factors on a 5-point Likert scale (0=not at all influential, 1=fairly uninfluential, 2=neutral, 3=fairly influential, 4=very influential). Each factor was assigned to one of five predefined domains: *Characteristics of the specialty*, *Personal and lifestyle considerations*, *Professional growth and interests*, *Previous experiences and interactions*, and *Demographic preferences*. For each respondent, a mean score for each domain was calculated, and group-level averages were computed for each specialty. These average domain scores were plotted using radar plots to visually profile differences in career priorities across specialties. Only final-year students were included in this analysis to minimise the influence of inexperience on specialty perceptions.

This study adheres to the Strengthening the Reporting of Observational Studies in Epidemiology (STROBE) guidelines.^29^

## Results

In total, 8,395 students representing all 44 UK medical schools participated in the survey. The mean response number per medical school was 191, and the median was 171 (IQR 124–224). A breakdown of the response numbers by medical school can be found in online supplemental material 4. The median age for participants was 22 (IQR 20–23). Although responses were obtained from all year groups, there were relatively fewer responses from students in the ‘Year 4 (not penultimate year)’ category, likely due to a smaller number of students presently enrolled in intercalated degrees or 6-year medical programmes rather than conventional 5-year curricula. Among the participants, 68.2% identified as female (n=5,727), 30.2% as male (n=2,537), 0.9% as non-binary (n=78) and 53 individuals preferred not to disclose their gender (table 1).

### Specialties

**Figure 1:**
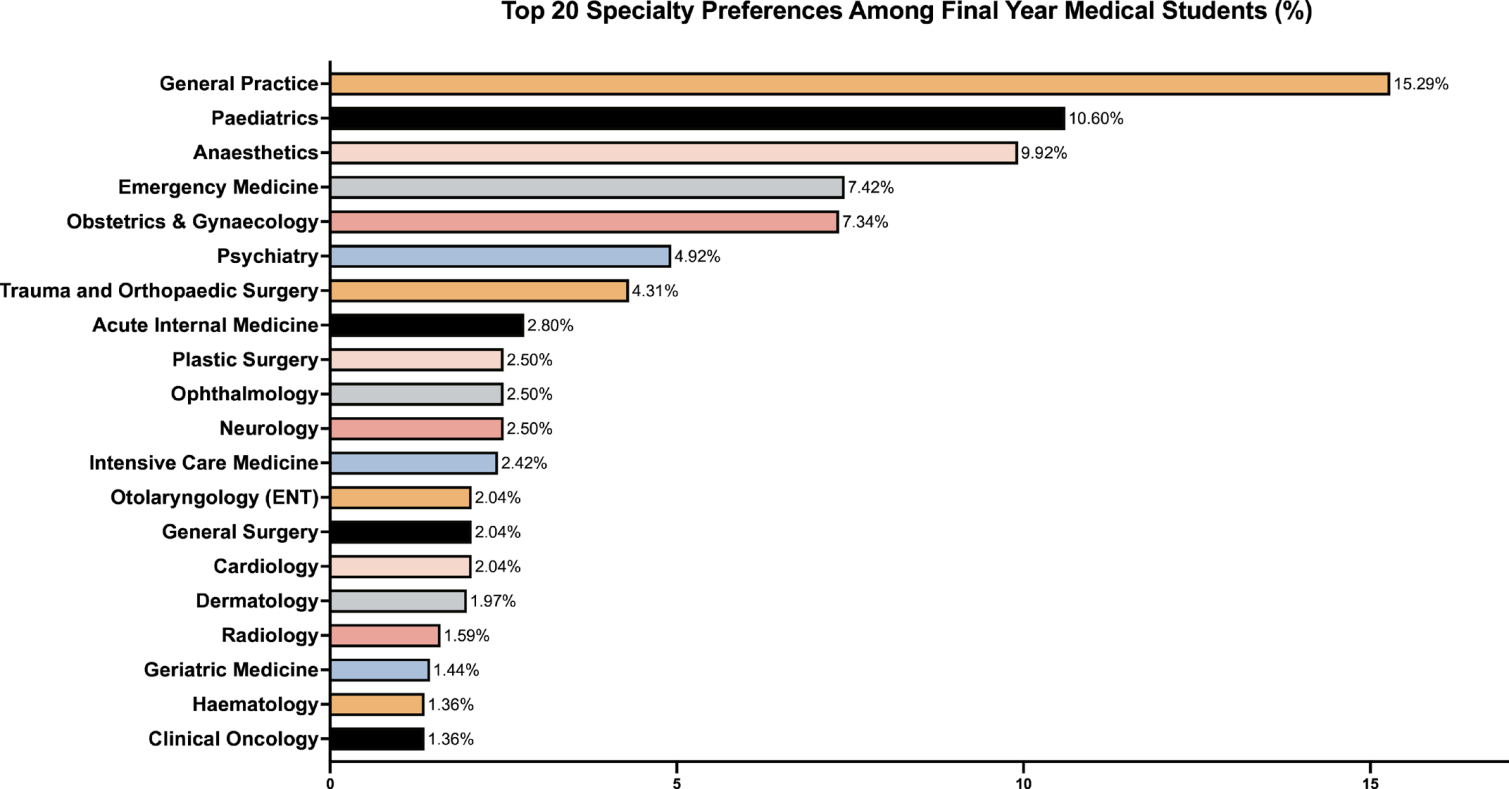
Top 20 specialty preferences among final-year medical students (%).

Survey respondents were asked to identify the specialty they are presently most interested in pursuing. Among all respondents, the ten most frequently cited specialties included General Practice (12.5%), Paediatrics (10.8%) and Emergency Medicine (8.3%). The most popular choices among final-year students were General Practice (15.3%), Paediatrics (10.6%) and Anaesthetics (9.9%). Figure 1 illustrates the distribution of final-year students’ specialty preferences, highlighting the relative popularity of each specialty. A complete breakdown, including data for first-year students and overall proportions, can be found in online supplemental material 5.

### Certainty and information

All participants were asked to express their level of certainty regarding which medical specialty they intend to pursue, as well as their perceived knowledge and understanding of the pathway towards this career. 6.5% of participants were very certain (n=547), 31.8% were fairly certain (n=2,667), 24.1% were neutral (n=2024), 23.8% were fairly uncertain (n=1,997) and 13.8% were very uncertain (n=1,160) (online supplemental material 6). 7.8% of students reported feeling fully informed and understanding of the pathway (n=654), 44.8% of students felt somewhat informed but needed more information (n=3,765), 11.7% of students felt neutral (n=980), 26.0% felt somewhat uninformed and unclear about the pathway and 9.7% of students felt not at all informed (n=812).

Multivariable logistic regression analysis revealed that male students were more likely to report being somewhat or very certain (OR 1.19, CI 1.07 to 1.31, p<0.0001) and well-informed (OR 1.30, CI 1.18 to 1.43, p<0.0001) about their specialty choices compared with female students, even after adjusting for demographic and educational background (online supplemental material 7). As expected, students in their final year were significantly more certain (OR 2.54, CI 2.14 to 3.02, p<0.0001) and better informed (OR 2.87, CI 2.41 to 3.41, p<0.0001) about their specialty preferences than earlier-year students. Students with a PubMed-indexed publication were also more certain (OR 1.40, CI 1.18 to 1.66, p<0.0001) and well-informed (OR 1.51, CI 1.25 to 1.82, p<0.0001) regarding their specialty choices.

### Confidence

The survey also assessed students’ confidence in securing specialty training posts amid increasing competition (14). Overall, only 23.1% (n=1,939) of participants reported feeling confident, while 40.4% (n=3,393) were neutral, and 36.5% (n=3063) lacked confidence. Confidence levels varied significantly across demographic groups (online supplemental material 8), including by ethnicity (χ²=35, df=4, p<0.0001). As with certainty of specialty choice, black students were most likely to express confidence (29.7%), whereas Asian students were least confident (20.1%) in securing a training place.

While controlling for demographic and educational predictors, male students were significantly more likely to report confidence in career progression than female students (OR 1.36, CI 1.21 to 1.52, p<0.0001). Compared with those from state comprehensive schools, confidence levels were similar among grammar school students (OR 1.02, CI 0.89 to 1.11, p=0.77) but significantly higher among privately educated students (OR 1.18, CI 1.03 to 1.35, p=0.02). Students with a physician parent or sibling also exhibited greater confidence in entering specialty training (OR 1.29, CI 1.13 to 1.47, p<0.0001). Additionally, confidence was notably higher among those who had completed an intercalated or previous degree (OR 1.22, CI 1.08 to 1.37, p<0.0001) and those with a PubMed-indexed publication (OR 1.67, CI 1.39 to 2.00, p<0.0001).

As shown in online supplemental table 11, confidence in securing specialty training became increasingly polarised among students further into their degrees. Even after adjusting for demographic and educational factors, final-year students were significantly more likely than first-year students to express confidence (OR 1.28, CI 1.05 to 1.56, p=0.02) but also more likely to be pessimistic about their chances (OR 1.62, CI 1.36 to 1.93, p<0.0001), suggesting a growing awareness of competition as students near graduation.

### Factors influencing choice of specialty

The survey quantitatively assessed a range of factors influencing students’ specialty choices. We grouped these factors into themes including work-life balance, financial considerations, career security, professional perception and prior experiences during training.

### Work-life balance and lifestyle considerations

Across all year groups, work-life balance emerged as the most influential factor, with 44.2% of students rating it as ‘fairly influential’ and 34.8% as ‘very influential’. Female students placed significantly more emphasis on this factor than males (OR 1.61, CI 1.43 to 1.79, p<0.0001), as well as stress and pressure at work (OR 1.92, CI 1.75 to 2.13, p<0.0001). Related considerations such as compatibility with family life (OR 1.41, CI 1.27 to 1.59, p<0.0001), out-of-hours demands (OR 1.47, CI 1.33 to 1.61, p<0.0001) and *preferences regarding working location (eg, in tertiary hospital vs district general hospital vs community)* (OR 1.15, CI 1.04 to 1.27, p=0.005) were also more valued by female students. Overall, compatibility with family life was a key determinant, rated as ‘fairly influential’ by 37.3% of students and ‘very influential’ by 39.4%.

### Financial considerations and career security

Overall, 66.1% of students considered financial remuneration an important factor in their specialty decision-making, while 40.7% were influenced by the potential for private practice earnings. Length of training (44.5%) and competition ratios (49.8%) were also key considerations. Compared with female students, males placed greater emphasis on remuneration (OR 1.11, CI 1.00 to 1.24, p=0.04) and private earnings (OR 1.52, CI 1.38 to 1.68, p<0.0001), while being less concerned about training length (OR 0.76, CI 0.69 to 0.84, p<0.0001) and competition ratios (OR 0.77, CI 0.69 to 0.84, p<0.0001).

Ethnic differences were also notable. Compared with white students, black students were significantly more likely to prioritise remuneration (OR 1.50, CI 1.26 to 1.79, p<0.0001), private earnings (OR 1.26, CI 1.06 to 1.50, p<0.0001), training length (OR 1.46, CI 1.23 to 1.73, p<0.0001) and competition ratios (OR 1.91, CI 1.61 to 2.26, p<0.0001). The differences were even more pronounced for Asian students, with corresponding ORs of 2.29 (CI 2.03 to 2.58, p<0.0001), 2.08 (CI 1.87 to 2.32, p<0.0001), 1.89 (CI 1.70 to 2.11, p<0.0001) and 1.82 (CI 1.64 to 2.03, p<0.0001), respectively.

Final-year students also placed significantly greater emphasis on remuneration (OR 1.50, CI 1.26 to 1.79, p<0.0001), private earnings (OR 1.26, CI 1.06 to 1.50, p<0.0001), training length (OR 1.46, CI 1.23 to 1.73, p<0.0001) and competition ratios (OR 1.91, CI 1.61 to 2.26, p<0.0001) compared with first-year students, again adjusting for other demographic and educational predictors.

### Professional perception and specialty prestige

Prestige was seemingly not a major driver for most students, with 56.5% rating it as ‘not at all influential’ or ‘fairly uninfluential’. However, the influence of specialty prestige was more pronounced among males than females (OR 1.58, CI 1.41 to 1.77, p<0.0001), and also significantly higher among Asian than white students (OR 1.63, CI 1.43 to 1.86, p<0.0001). Stereotypes surrounding specialties were also of limited concern, with 60.3% considering them ‘not at all influential’ or ‘fairly uninfluential’. Role models and mentorship, by contrast, played an important role, with 55% of students rating them as ‘fairly’ or ‘very’ influential.

### Academic and intellectual influences

Intellectual challenge was a strong motivator for 67.4% of students, while 37.8% considered research opportunities ‘fairly influential’. The use of advanced technology in a specialty was less frequently cited as a key factor, with 31.5% rating it as ‘fairly influential’. These considerations were much more important for students with previous publications than those without, as evidenced by estimated ORs of 1.44 (CI 1.18 to 1.75, p<0.0001), 2.52 (CI 2.11 to 3.00, p<0.0001) and (OR 1.42, CI 1.18 to 1.71, p<0.0001), respectively. Males also valued research opportunities (OR 1.24, CI 1.12 to 1.37, p<0.0001) and use of technology (OR 2.08, CI 1.88 to 2.31, p<0.0001) more highly than female students.

### Clinical and training experiences

Past experiences during medical training strongly influenced specialty preferences. Positive interactions during rotations or clinical placements were considered ‘fairly influential’ by 38.6% and ‘very influential’ by a further 33.4%. Patient interaction and diversity of cases were also major considerations, with 77.4% and 64.6% of students, respectively, rating these factors as important. Female students were significantly more influenced by previous experiences (OR 1.3, CI 1.19 to 1.43, p<0.0001) and patient interaction (OR 2.27, CI 2.04 to 2.56, p<0.0001), as well as the opportunity to work with specific gender groups (OR 2.27, CI 1.89 to 2.7, p<0.0001) and specific age groups (OR 1.85, CI 1.67 to 2.08, p<0.0001) than their male counterparts.

### Gender and representation in specialty choices

The gender distribution of doctors within a specialty was not a strong factor for most students, with 50% finding it ‘not at all influential’ or ‘fairly uninfluential’. However, female students were considerably more influenced by this than male students (OR 3.57, CI 3.12 to 4.17, p<0.0001), suggesting that representation within a specialty may play a role in career decision-making for some groups.

Table 2 presents the factors influencing specialty training preferences among final-year students, indicating their importance on a Likert scale ranging from ‘very important’ to ‘not at all important.’ The table shows the proportion of students who selected each level of importance for each factor. Aggregate data for all year groups and specific data for first-year students are available in onlinesupplemental materials 9  10, respectively.

**Figure 2:**
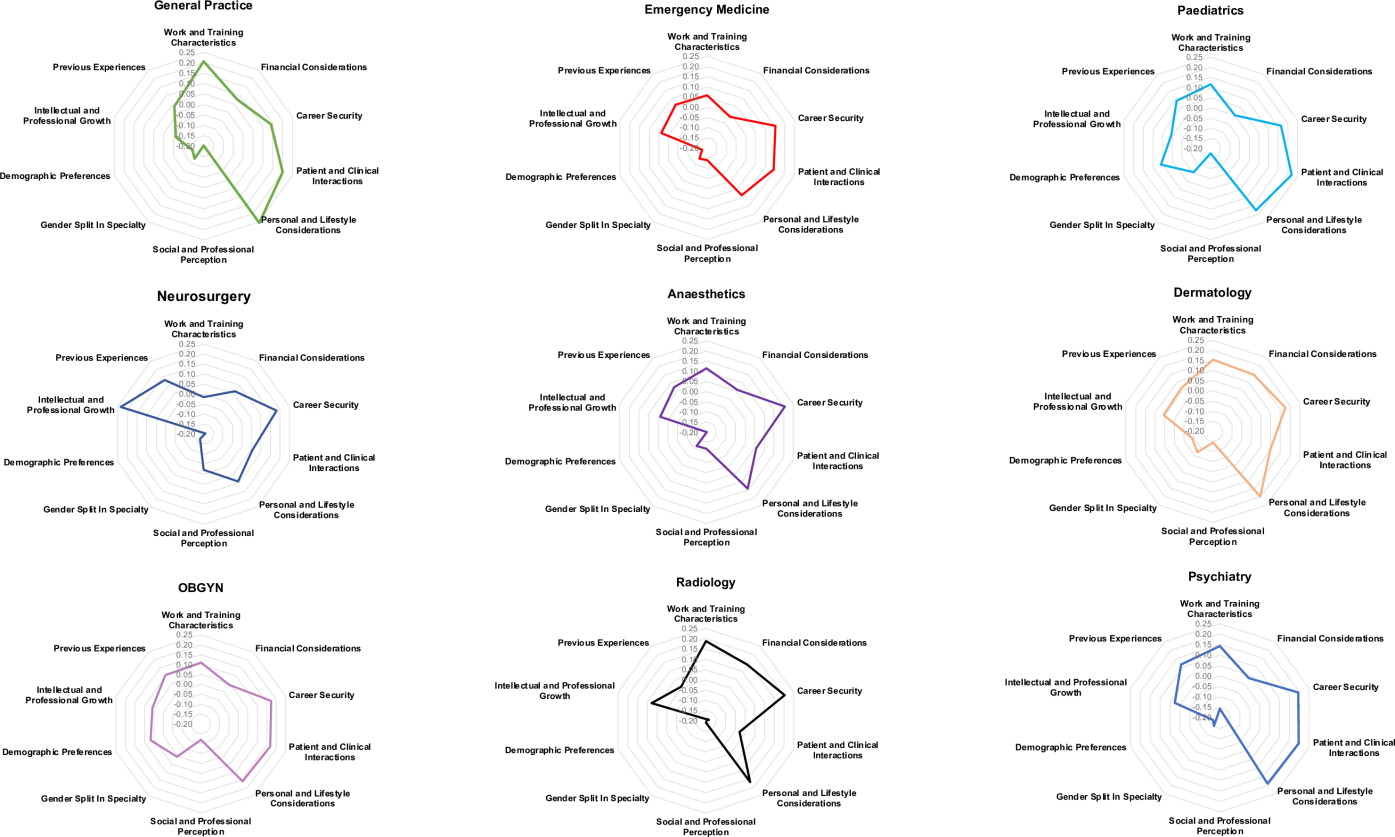
Specialty-specific factors influencing career choice among final-year medical students. Each student rated 28 individual factors on a 5-point Likert scale (not at all influential=0, fairly uninfluential=1, neutral=2, fairly influential=3, very influential=4). These factors were grouped into five domains: (1) characteristics of the specialty; (2) personal and lifestyle considerations; (3) professional growth and interests; (4) previous experiences and interactions; (5) demographic preferences. For each student’s selected specialty, a mean score for each domain was calculated, producing a specialty-specific profile of priorities. Radar plots illustrate the average importance assigned to each domain by students pursuing different specialties.

To better understand the factors influencing medical students’ specialty choices, these factors were grouped into ten distinct domains, each representing a broader category of related considerations. These data were used to generate radar plots visually representing the relative importance of each domain to students pursuing different specialties. A visual summary of the relative importance of grouped factors for selected specialties is shown in figure 2, focusing on final-year students, who are likely better informed regarding nuances of each specialty.

## Discussion

### Key findings

This study, the largest of its kind, explored the factors influencing specialty preferences among UK medical students. Lifestyle factors such as work-life balance and financial remuneration emerged as primary factors, corroborating existing literature.^7 9 30^ Significant disparities were observed in certainty of career choice, confidence in entering the profession, and knowledge about specialty pathways across multiple demographic groups. Notably, there was an overwhelming lack of confidence among students of all years regarding their ability to secure a specialty training place, likely influenced by current training bottlenecks and competition ratios.^5 6^

### Certainty

Our findings indicate rising certainty among students regarding their preferred specialty as they progress through medical school. Although this number appears remarkably low, it has previously been demonstrated that even qualified doctors feel rushed into choosing their career pathway, with their specialty certainty rising with each year post-qualification.^31 32^ More conscious efforts should be made by medical schools to formalise career guidance and captivate early interest in specialties, enabling students to begin to prepare for increasingly competitive postgraduate training application processes.^5 6^

Males reported higher certainty than females in their choice of specialty. This could perhaps reflect the male-dominated state of many specialties driving female students to uncertainty.^33 34^ Furthermore, gender biases within medical education and professional environments may reinforce these traits in males, providing them with more encouragement and validation. Male medics may also experience fewer societal or in-work expectations regarding work-life balance, allowing them to focus more on their career paths without the added pressure of balancing family and professional responsibilities.^34^ Less-than-full-time (LTFT) training, an extension of specialty training duration to support work-life balance, has been a useful mechanism to support female specialty trainees to overcome this barrier. However, despite LTFT’s increasing popularity among specialty trainees of all genders and backgrounds, stigma persists (particularly in surgical specialties) and thus limits its uptake.^35 36^ This combination of cultural conditioning, reinforcement of confidence and fewer perceived barriers likely contributes to males being more certain about their specialty choices.

### Confidence

Regarding confidence in securing a specialty training position in their specialty of choice, males were more likely to express confidence than females, again perhaps due to gender biases that can lead to more positive reinforcement for males, and a greater visibility of male role models in certain specialties.^37^

Our analysis finds that confidence in securing a specialty training post correlates more closely with extracurricular achievements than with holding a previous degree (ref). This aligns with the current structure of specialty training applications, where extracurricular achievements such as publications, leadership roles and awards are increasingly used as differentiating factors. The recent removal of recognition for intercalated degrees or those obtained prior to studying medicine from scoring criteria only reinforces the importance of these achievements.^38^ Hence, students may feel greater pressure to accumulate such accolades during medical school, potentially exacerbating disparities among those with differing access to opportunities.

### Physician parent or sibling

Although students with a physician parent or sibling were more likely to feel confident in their ability to obtain a training post, they were generally less certain about their choice of specialty compared with those without. These students, on account of this familial factor, might be benefitting from increased guidance, insight and networking opportunities, which could boost confidence in navigating the competitive training landscape. Additionally, as we highlight in a separate analysis of FAST study data, these students are significantly more likely to accrue extracurricular accolades relevant to specialty applications, namely, publications, which could account for increased confidence levels (ref). However, exposure to the diverse challenges and demands of various specialties through their family members could also result in a more realistic understanding of certain fields, leading to greater deliberation and uncertainty in making a definitive specialty choice.

### Specialty preferences among all students

The specialty preferences among all medical students reflect a diverse range of interests, with General Practice (12.5%), Paediatrics (10.8%) and Emergency Medicine (8.3%) being the three most popular. This distribution suggests a significant interest in generalist specialties that offer broad patient interactions and diverse clinical challenges. It also highlights the popularity of General Practice; however, this falls extremely short of the 50% target set by the Department for Health.^39 40^ The proportion of students indicating a preference for General Practice as a career increased from 8.6% among first years to 15.3% among final-year medical students. It is probable that the figure would be even higher if early-year doctors were included in the study, given that General Practice comprised 34.1% of training places for 2024 entry.^5^ This may reflect a growing recognition of the need for primary care physicians, a pragmatic response to high competition ratios in other specialties, or a shift towards favouring specialties which may be seen to offer a better work-life balance. Moreover, this increase in preference for General Practice could be seen as a consequence of the broader systemic push towards creating a workforce of ‘generalist’ doctors. This strategy aligns with the vision articulated by England’s Chief Medical Officer, who has vocalised a need to have doctors with broad-based skills to meet the future needs of the NHS.^41^ The concept of ‘GP factories’—medical schools or programmes specifically designed to produce a high proportion of General Practitioners—is becoming increasingly relevant in this context.^41 42^

Interestingly, high-competition specialties such as Neurosurgery (4.3% in Year 1 vs 0.61% final year) and Cardiothoracic Surgery (4.1% overall vs 0.61% final year) show a decrease in preference among final-year students. This could indicate a greater understanding of the feasibility and desirability of pursuing these demanding fields as students near the end of their training. Students’ early enthusiasm for these specialties may reflect societal perceptions and media portrayals of the specialties.^43^

### Factors affecting career choice

The notable gender differences in specialty preference factors suggest varying priorities between male and female students. Females’ higher concern for work-life balance, stress and family compatibility could be linked to societal expectations and potential caregiving responsibilities. These preferences are consistent with previous research indicating that female doctors gravitate towards specialties offering more predictable hours and better work-life balance.^44 45^ Further, females’ greater regard for the gender distribution of doctors within specialties could indicate a desire for role models and a supportive working environment that aligns with their demographic.^37^ Financial considerations, including private practice opportunities, were moderately influential. The significant difference in how males and females value financial remuneration could reflect varying career expectations and economic motivations. Females’ higher concern about the number of examinations and training costs could suggest a pragmatic approach to the financial and time investment required for specialty training. The lower concern for competition ratios among privately schooled students might suggest greater confidence in navigating competitive environments, potentially due to better preparatory resources and support networks.

### Specialty-specific influencing factors

The plots in figure 2 illustrate the contrasting priorities of final-year students favouring different specialties, in terms of the relative importance of various factors. For instance, General Practice and Paediatrics attract students who place significant emphasis on lifestyle compatibility and direct patient care. On the other hand, those interested in Neurosurgery and Cardiothoracic Surgery are particularly driven by intellectual challenge and opportunities for professional growth. The radar plots also reveal distinct variations in the perceived importance of financial considerations and career security across specialties. For example, students inclined towards Dermatology and Anaesthetics were especially influenced by financial remuneration, possibly reflecting perceptions of higher private earning potential in these fields.

Overall, these visualisations emphasise the diverse motivations driving specialty preferences. By acknowledging these varied priorities, medical educators and policymakers can offer more tailored career advice to better support students in their decision-making. This will ultimately contribute to a more satisfied and effective workforce in which future doctors are well-matched to their specialties.

### Broader context and implications

The FAST study reveals alarmingly low levels of confidence among medical students, particularly in securing specialty training posts. Lack of confidence, even among final-year students, hints at growing disillusionment with the training process and perhaps a reflection of current competition ratios.^5^ Graduates increasingly unable to enter training may opt to emigrate, given that more than one-third of students already intend to exit the NHS.^7^ While increasing medical school places may be seen by some as a step towards progress, it is not a panacea, for without addressing issues highlighted here and elsewhere, these efforts will not succeed. This trend has damaging implications for the NHS, exacerbating existing workforce shortages and increasing reliance on internationally trained doctors.^46 47^ It also reinforces that merely increasing medical school places is insufficient to address the broader issue of retention if systemic issues within training, education and career progression are not addressed.

Medical schools have a vital role in shaping students’ career intentions, highlighting the importance of questionnaire-based workforce planning in healthcare.^8^ By gaining insights into students’ career trajectories and the factors influencing their specialty choices, medical schools can better align their programmes to meet the needs of both students and the NHS. This proactive approach is crucial for developing a dynamic and responsive healthcare system.^8^ Students’ career intentions will need to be regularly monitored in future, building on the AIMS and FAST studies.^7^ This will help anticipate future training bottlenecks and better align the supply and demand for specialists, ultimately contributing to a more sustainable healthcare system.

Our findings suggest that the government’s strategy of producing ‘pluripotent’ doctors^48^—medical graduates who remain generalists—might be inadvertently succeeding. Increasing uncertainty among medical students about their specialty choices could lead to a generation of doctors who struggle to specialise. Despite possibly boosting short-term workforce flexibility, this risks limiting access to specialists in the future, and leading to dissatisfaction with service provision, potentially affecting the quality of care and long-term retention within the NHS. Further, the ‘lost tribe’ of doctors—those stuck in non-training, service-only roles despite their qualifications—highlights a growing crisis in the UK medical workforce.^49 50^ This issue, exacerbated by highly competitive training applications and diminished recognition of prior academic achievements, leaves many junior doctors unable to progress in their careers. Our findings illustrate low confidence among medical students in securing specialty training posts, even in those in their final years, which suggests that more graduates expect to join this ‘lost tribe’. This trend is contributing to an overburdened workforce increasingly reliant on alternative routes like the Certificate of Eligibility for Specialist Registration, or portfolio pathway and informal training schemes.^51^ Addressing this issue is crucial for ensuring that the NHS can effectively train and retain its doctors, rather than creating a parallel workforce of underutilised professionals.

### Recommendations

#### Address bottlenecks in career progression

The increasing competition for specialty posts has created marked uncertainty among students, a sentiment reportedly shared by many early-career doctors.^52 53^ Expanding training places and improving transparency in selection criteria will help align student aspirations with workforce needs. Addressing these bottlenecks is critical for ensuring workforce sustainability.

#### Expand and destigmatise LTFT training

LTFT training should be expanded across all specialties and normalised as a viable option for all doctors. It is important to clarify that LTFT often refers to a 40-hour working week, which is considered full-time in other employment settings. Given that work-life balance is a significant concern for many medical students, expanding and normalising LTFT training can make demanding specialties more accessible, reduce burnout and ultimately help retain more doctors within the NHS.^35 36^

#### Improve career guidance in medical schools

Medical schools should provide structured, evidence-based career advice beginning in the early years of training. This should include formalised and dynamic mentorship schemes, clear and regularly updated resources on specialty application processes, and early exposure to specialty portfolio requirements. Beyond standard specialty placements, dedicated ‘taster weeks’ in clinical years should be introduced, allowing students to step away from their core curriculum to gain deeper insight into their specialties of interest. These experiences would enable students to build meaningful connections with role models, fostering mentorship and long-term career support.

Of course, no amount of career advice can compensate for the reality of extreme competition ratios in certain specialties. However, ensuring students have not only more time to build their portfolios but also a clearer framework for doing so will maximise their chances of success. Early, structured guidance would allow students to identify key requirements well in advance, enabling them to develop competitive applications while making informed and strategic decisions about their future careers.

#### Ensure equitable access to opportunities

Differences in socioeconomic background impact access to research, leadership and academic opportunities. Medical schools should offer structured support, such as funded research placements and mentoring, to reduce disparities.

### Limitations

This study is not without its limitations. The cross-sectional design restricts our ability to infer causality, and future longitudinal studies are needed to explore how the observed factors interact over time. Self-reported data may introduce recall and social desirability biases. Participants, particularly early-year students who may have limited exposure to different specialties, may have provided responses on topics they are not yet fully familiar with, potentially affecting the reliability of the data. Socioeconomic status was inferred through educational background rather than direct financial measures, which may not fully capture its impact. More intrusive but direct measures could have provided clearer insights but were avoided to maintain participant engagement. Academic performance data were not collected, which may have provided additional context on specialty confidence and career aspirations.

Although key demographic and educational variables were accounted for, residual confounding remains possible due to unmeasured factors beyond the scope of this study. Additionally, as survey participation was voluntary and distributed predominantly online, the response rate is unknown. Finally, these findings are specific to UK medical students and may not be directly applicable to other healthcare systems with different training structures and workforce pressures.

## Conclusion

This study highlights significant disparities in specialty preferences and the factors influencing these decisions among UK medical students, emphasising the impact of demographics, extracurricular participation and specialty exposure on career trajectories. Notably, General Practice, Paediatrics and Anaesthetics were among the most preferred specialties. Work-life balance, compatibility with family life and specialty outlook were consistently the most influential factors, particularly for final-year students. Levels of certainty and confidence varied considerably by gender, ethnicity and educational background, highlighting the need for tailored support to ensure equitable career progression. Medical schools and policymakers must prioritise improving career guidance and expanding early exposure to a diverse range of specialties, and advocate for an increase in specialty training posts. Addressing these factors is crucial for cultivating a motivated and sustainable medical workforce that meets the evolving needs of the NHS. Without targeted interventions, these disparities risk perpetuating bottlenecks in training and further talent loss from the healthcare system.

## Supplementary material

10.1136/bmjopen-2025-103061 online supplemental material 1

10.1136/bmjopen-2025-103061 online supplemental material 2

10.1136/bmjopen-2025-103061 online supplemental material 3

10.1136/bmjopen-2025-103061 online supplemental material 4

10.1136/bmjopen-2025-103061 online supplemental material 5

10.1136/bmjopen-2025-103061 online supplemental material 6

10.1136/bmjopen-2025-103061 online supplemental material 7

10.1136/bmjopen-2025-103061 online supplemental material 8

10.1136/bmjopen-2025-103061 online supplemental material 9

10.1136/bmjopen-2025-103061 online supplemental material 10

10.1136/bmjopen-2025-103061 online supplemental material 11

## Data Availability

All data relevant to the study are included in the article or uploaded as supplemental information.
