# Supplementary material for: Specialty choices among UK medical students: certainty, confidence and key influences—a national survey (FAST Study)
Source: BMJ Open. 2025 Aug 8;15(8):e103061. doi: 10.1136/bmjopen-2025-103061 (PMC12336620; doi:10.1136/bmjopen-2025-103061)
Supplement: online supplemental material 1 [file bmjopen-15-8-s001.pdf]

FAST STUDY

Factors Affecting Specialty Training preference among UK medical students

University of BRISTOL

Demographics

FAST - Factors Affecting Specialty Training preference among UK medical students: a national cross-sectional survey

Thank you for taking part in the study. Please note that participating in this survey is *entirely optional.*

FAST seeks to understand the factors influencing UK medical students' choice of specialty. We aim to identify key determinants in career decision-making and gather insights into the preferences and expectations surrounding specialty choice.

All responses will remain confidential. Your email address will only be visible to the study leads and will be deleted from our records once all data has been collected and there is no need for further communication. You may withdraw from the study at any point by contacting Tomas Ferreira, **tf385@cam.ac.uk**.

By submitting your answers to the survey, you consent to us collecting this data and acknowledging that anonymised data may be published and used for purposes beyond this study.

Please read the Participant Information Sheet (PIS) which can be found here.

The survey should take an average of 6 minutes to complete, and all participants will be entered into a prize draw for the chance to win ***£250***!

I understand that my participation is voluntary and that I am free to withdraw at any time without giving a reason and I consent to participate in this study. Participant Information Sheet available here.

○ Yes

Email Address (ending in 'ac.uk')

Please enter your institutional email address (ending in 'ac.uk'. We will use this to verify your student status and we may contact you to notify you of a prize win). This will be deleted once all data has been collected and not linked to your responses in any way.

Please ensure there are no spaces at the end of your email.

Duplicate entries and responses with non-institutional email addresses will be deleted to ensure integrity of results.

Age

Gender

○ Female
○ Male

- ○ Non-binary
- ○ Prefer not to say

Which of the below options best describes your ethnicity?

[ ]

University

[ ]

Year of study - Please read description.

(as of September 2023)

- If you are in your fourth year of study and it is your final year, please select final year (i.e., GEM)

- If you are in your fourth year of study but it is your penultimate year, please select penultimate year.

- If you are currently intercalating, please select your current year of study (e.g., intercalating between 3rd and 4th year on a 5 year course please select Year 4).

- Treat the first and second year of a GEM course as still equivalent to first and second year.

- If you are in a "Foundation" or "Gateway" year (also known as Y0), please select Year 1.

- Disregard resits, select the year group of your cohort.

- ○ Year 1
- ○ Year 2
- ○ Year 3 **(but not penultimate year)**
- ○ Year 4 **(but not penultimate or final year)**
- ○ Penultimate year
- ○ Final year

What is your expected graduation year?

[ ]

Do you have a previous or intercalated degree?

- ○ Yes, a degree prior to studying Medicine.

- ◯ Yes, an intercalated degree.
- ◯ Yes, both.
- ◯ Not yet, but intend on intercalating.
- ◯ Not yet, but currently intercalating.
- ◯ No.

What is your student fee status?

- ◯ Home
- ◯ EU/EEA
- ◯ International (Non-EU)

Which option *best* describes your schooling?

- ◯ Comprehensive state school
- ◯ Selective state school or grammar school
- ◯ Private school (fee-paying)
- ◯ Prefer not to say

Do you have a parent or a sibling who is/was a doctor?

- ◯ Yes
- ◯ No

Do you have any of the following? (tick all that apply)

- ☐ First author on a PubMed-indexed publication
- ☐ Author on a PubMed-indexed publication
- ☐ Cited Collaborative author on a PubMed-indexed publication
- ☐ Involvement in an audit/QI project
- ☐ Poster presentation
- ☐ Oral presentation
- ☐ National or international prize related to Medicine (e.g., essay or conference prizes)
- ☐ National leadership role
- ☐ Regional or local leadership role

- [ ] Medical school examination merit/prize(s)
- [ ] None of the above

Specialty Preferences

How certain are you about your current choice of specialty?

- ○ Very certain
- ○ Fairly certain
- ○ Neutral
- ○ Fairly uncertain
- ○ Very uncertain

Which medical specialty are you most interested in currently?

If you are unsure, or not set on one specialty, please select the one you are favouring the most currently.

We will take under consideration your uncertainty level from the previous question when analysing responses.

- ○ Acute Internal Medicine
- ○ Allergy
- ○ Anaesthetics
- ○ Audio Vestibular Medicine
- ○ Aviation and Space Medicine
- ○ Cardiothoracic Surgery
- ○ Cardiology
- ○ Clinical Genetics
- ○ Clinical Neurophysiology
- ○ Clinical Oncology
- ○ Chemical Pathology (Metabolic Medicine/Clinical Biochemistry)
- ○ Clinical Pharmacology and Therapeutics
- ○ Dermatology
- ○ Diagnostic Neuropathology
- ○ Emergency Medicine
- ○ Endocrinology and Diabetes

- ○ Gastroenterology
- ○ General Practice
- ○ General Surgery
- ○ Geriatric Medicine
- ○ Genitourinary Medicine
- ○ Haematology
- ○ Histopathology
- ○ Immunology
- ○ Infectious Disease (inc. Microbiology, Virology, Tropical Medicine)
- ○ Intensive Care Medicine
- ○ Medical Oncology
- ○ Neurology
- ○ Neurosurgery
- ○ Nuclear Medicine
- ○ Obstetrics & Gynaecology
- ○ Occupational Health
- ○ Oral and Maxillofacial Surgery
- ○ Ophthalmology
- ○ Otolaryngology (ENT)
- ○ Pathology
- ○ Paediatric Cardiology
- ○ Paediatric Surgery
- ○ Paediatrics
- ○ Palliative Medicine
- ○ Pharmaceutical Medicine
- ○ Plastic Surgery
- ○ Psychiatry
- ○ Radiology
- ○ Rehabilitation Medicine
- ○ Renal Medicine
- ○ Respiratory Medicine
- ○ Rheumatology
- ○ Sexual and Reproductive Health (CSRH)
- ○ Sport and Exercise Medicine
- ○ Trauma and Orthopaedic Surgery

- ○ Urology
- ○ Vascular Surgery

Do you feel adequately informed about the pathway to pursue your chosen specialty in the UK?

- ○ Fully informed and understand the pathway
- ○ Somewhat informed, but need more details
- ○ Neutral
- ○ Somewhat uninformed and unclear about the pathway
- ○ Not informed at all

In the present context, how confident are you in your chances of securing a training post in your chosen specialty in the UK?

- ○ Very confident
- ○ Fairly confident
- ○ Neutral
- ○ Fairly unconfident
- ○ Not confident at all

Factors affecting specialty choice

How important are the following factors in influencing your choice of specialty? (1/2)

	Not at all influential	Fairly uninfluential	Neutral	Fairly Influential	Very influential
Work-life balance	○	○	○	○	○
Financial remuneration	○	○	○	○	○
Potential for private practice earnings	○	○	○	○	○
Length of specialty training	○	○	○	○	○

Level of stress and pressure at work	○	○	○	○	○
Level of competition for entry into the specialty	○	○	○	○	○
Future outlook of the specialty	○	○	○	○	○
Training structure (run through i.e., entry at ST1 vs. uncoupled i.e., entry via core training and having to reapply at ST3)	○	○	○	○	○
Number of exams and overall cost of specialty training	○	○	○	○	○
Continuity of care with patients	○	○	○	○	○
Compatibility with family life	○	○	○	○	○
Out-of-hours demands (OOH shifts)	○	○	○	○	○
Geographic location preference (e.g., tertiary hospital vs district general hospital vs community)	○	○	○	○	○
Perceived prestige of specialty	○	○	○	○	○
Stereotypes surrounding specialty	○	○	○	○	○
Influence of mentors or role models	○	○	○	○	○

How important are the following factors in influencing your choice of specialty? (2/2)

	Not at all influential	Fairly uninfluential	Neutral	Fairly Influential	Very influential
Intellectual challenge	○	○	○	○	○
Research opportunities within specialty	○	○	○	○	○

Use of advanced technology in the specialty	○	○	○	○	○
Use of clinical diagnostic skills vs. investigations	○	○	○	○	○
Interest in specific conditions	○	○	○	○	○
Personal experiences of disease	○	○	○	○	○
Pre-clinical positive experiences with the specialty (e.g., lectures, tutorials)	○	○	○	○	○
Past positive interactions with the specialty (e.g., rotations, clinical attachments)	○	○	○	○	○
Preference for working with specific gender groups	○	○	○	○	○
Preference for working with specific age groups (e.g., geriatrics, paediatrics)	○	○	○	○	○
Level of patient interaction	○	○	○	○	○
Diversity of patient interactions	○	○	○	○	○
Gender distribution of doctors in the specialty	○	○	○	○	○

Are there any *other* factors that significantly influence your choice of specialty? (Optional)

Powered by Qualtrics
