# Supplementary material for: Specialty choices among UK medical students: certainty, confidence and key influences—a national survey (FAST Study)
Source: BMJ Open. 2025 Aug 8;15(8):e103061. doi: 10.1136/bmjopen-2025-103061 (PMC12336620; doi:10.1136/bmjopen-2025-103061)
Supplement: online supplemental material 5 [file bmjopen-15-8-s005.docx]

| **Population** | **Specialty** | **Count** | **%** |
| --- | --- | --- | --- |
| Overall |  |  |  |
|  | General Practice | 1,047 | 12.5% |
|  | Paediatrics | 909 | 10.8% |
|  | Emergency Medicine | 700 | 8.3% |
|  | Obstetrics & Gynaecology | 603 | 7.2% |
|  | Anaesthetics | 505 | 6.0% |
|  | Trauma and Orthopaedic Surgery | 460 | 5.5% |
|  | Psychiatry | 442 | 5.3% |
|  | Cardiology | 327 | 3.9% |
|  | Dermatology | 278 | 3.3% |
|  | General Surgery | 254 | 3.0% |
|  | Neurology | 235 | 2.8% |
|  | Plastic Surgery | 224 | 2.7% |
|  | Neurosurgery | 212 | 2.5% |
|  | Ophthalmology | 180 | 2.1% |
|  | Cardiothoracic Surgery | 160 | 1.9% |
|  | Acute Internal Medicine | 134 | 1.6% |
|  | Radiology | 129 | 1.5% |
|  | Clinical Oncology | 116 | 1.4% |
|  | Paediatric Surgery | 114 | 1.4% |
|  | Otolaryngology (ENT) | 112 | 1.3% |
| Year 1 |  |  |  |
|  | Paediatrics | 145 | 11.0% |
|  | General Practice | 114 | 8.6% |
|  | Emergency Medicine | 111 | 8.4% |
|  | Obstetrics & Gynaecology | 80 | 6.1% |
|  | Trauma and Orthopaedic Surgery | 75 | 5.7% |
|  | Cardiology | 73 | 5.5% |
|  | Dermatology | 64 | 4.8% |
|  | Psychiatry | 63 | 4.8% |
|  | Neurosurgery | 57 | 4.3% |
|  | Cardiothoracic Surgery | 54 | 4.1% |
|  | Anaesthetics | 45 | 3.4% |
|  | General Surgery | 39 | 3.0% |
|  | Plastic Surgery | 35 | 2.7% |
|  | Neurology | 29 | 2.2% |
|  | Sport and Exercise Medicine | 27 | 2.0% |
|  | Clinical Oncology | 22 | 1.7% |
|  | Ophthalmology | 21 | 1.6% |
|  | Paediatric Surgery | 21 | 1.6% |
|  | Radiology | 19 | 1.4% |
|  | Medical Oncology | 14 | 1.1% |
