# Supplementary material for: Specialty choices among UK medical students: certainty, confidence and key influences—a national survey (FAST Study)
Source: BMJ Open. 2025 Aug 8;15(8):e103061. doi: 10.1136/bmjopen-2025-103061 (PMC12336620; doi:10.1136/bmjopen-2025-103061)
Supplement: online supplemental material 6 [file bmjopen-15-8-s006.docx]

| **Characteristic** | | **Very certain** | **Fairly certain** | **Neutral** | **Fairly uncertain** | **Very uncertain** |
| --- | --- | --- | --- | --- | --- | --- |
| *Ethnicity* | |  |  |  |  |  |
|  | Asian or Asian British | 6.2% | 28.9% | 27.9% | 24.0% | 13.0% |
|  | Black, Black British, Caribbean or African | 8.6% | 33.8% | 25.3% | 20.2% | 12.2% |
|  | Mixed or multiple ethnic groups | 8.5% | 26.6% | 22.8% | 24.6% | 17.4% |
|  | White | 5.9% | 34.1% | 21.7% | 24.1% | 14.1% |
|  | Other | 9.6% | 28.5% | 24.3% | 24.6% | 13.0% |
|  | Prefer not to say | 11.3% | 23.7% | 33.0% | 15.5% | 16.5% |
| *Gender* | |  |  |  |  |  |
|  | Female | 5.6% | 31.5% | 23.2% | 24.9% | 14.8% |
|  | Male | 8.3% | 32.3% | 26.1% | 21.4% | 11.8% |
|  | Non-binary | 11.5% | 41.0% | 20.5% | 20.5% | 6.4% |
|  | Prefer not to say | 15.1% | 24.5% | 28.3% | 20.8% | 11.3% |
| *Level of education* | |  |  |  |  |  |
|  | Postgraduate | 8.7% | 36.9% | 24.9% | 20.8% | 8.7% |
|  | Undergraduate | 6.0% | 30.6% | 23.9% | 24.4% | 14.9% |
| *Previous schooling* | |  |  |  |  |  |
|  | Comprehensive state school | 6.3% | 32.1% | 24.0% | 23.8% | 13.9% |
|  | Selective state school or grammar school | 5.9% | 31.3% | 23.2% | 25.4% | 14.2% |
|  | Private school (fee-paying) | 7.4% | 33.0% | 24.0% | 21.9% | 13.6% |
|  | Prefer not to say | 7.4% | 19.9% | 33.6% | 27.7% | 11.4% |
| *Parent or sibling in Medicine* | |  |  |  |  |  |
|  | Yes | 6.9% | 29.4% | 23.9% | 25.0% | 14.8% |
|  | No | 6.4% | 32.4% | 24.2% | 23.5% | 13.6% |
| *Fee status* | |  |  |  |  |  |
|  | Home | 6.0% | 31.5% | 23.7% | 24.3% | 14.6% |
|  | EU/EEA | 10.6% | 35.8% | 21.5% | 20.8% | 11.3% |
|  | International (non-EU) | 10.2% | 32.9% | 28.5% | 20.6% | 7.9% |
| *Year of study* | |  |  |  |  |  |
|  | Year 1 | 4.5% | 25.5% | 30.7% | 24.5% | 14.8% |
|  | Year 2 | 4.6% | 28.8% | 24.3% | 27.3% | 15.1% |
|  | Year 3 (but not penultimate year) | 4.3% | 27.5% | 26.0% | 25.5% | 16.6% |
|  | Year 4 (but not penultimate or final year) | 6.7% | 29.1% | 26.5% | 22.9% | 14.8% |
|  | Penultimate year | 7.7% | 36.0% | 20.7% | 22.4% | 13.2% |
|  | Final year | 11.9% | 42.8% | 18.4% | 19.0% | 7.9% |
