# Supplementary material for: Specialty choices among UK medical students: certainty, confidence and key influences—a national survey (FAST Study)
Source: BMJ Open. 2025 Aug 8;15(8):e103061. doi: 10.1136/bmjopen-2025-103061 (PMC12336620; doi:10.1136/bmjopen-2025-103061)
Supplement: online supplemental material 7 [file bmjopen-15-8-s007.docx]

| **Characteristic** | | **Fully informed and understand the pathway** | **Somewhat informed, but need more details** | **Neutral** | **Somewhat uninformed and unclear about the pathway** | **Not informed at all** |
| --- | --- | --- | --- | --- | --- | --- |
| *Ethnicity* | |  |  |  |  |  |
|  | Asian or Asian British | 6.5% | 42.5% | 13.7% | 27.3% | 9.9% |
|  | Black, Black British, Caribbean or African | 9.6% | 48.1% | 9.2% | 22.0% | 11.2% |
|  | Mixed or multiple ethnic groups | 9.4% | 42.7% | 8.9% | 28.2% | 10.7% |
|  | White | 8.0% | 46.8% | 10.4% | 25.7% | 9.1% |
|  | Other | 9.0% | 39.3% | 16.9% | 24.6% | 10.2% |
|  | Prefer not to say | 11.3% | 29.9% | 23.7% | 20.6% | 14.4% |
| *Gender* | |  |  |  |  |  |
|  | Female | 5.8% | 44.9% | 11.3% | 27.6% | 10.3% |
|  | Male | 11.9% | 44.8% | 12.3% | 22.6% | 8.4% |
|  | Non-binary | 11.5% | 48.7% | 10.3% | 24.4% | 5.1% |
|  | Prefer not to say | 15.1% | 35.8% | 18.9% | 15.1% | 15.1% |
| *Level of education* | |  |  |  |  |  |
|  | Postgraduate | 13.0% | 48.5% | 7.8% | 22.4% | 8.2% |
|  | Undergraduate | 6.6% | 44.0% | 12.5% | 26.8% | 10.0% |
| *Previous schooling* | |  |  |  |  |  |
|  | Comprehensive state school | 7.7% | 45.5% | 11.5% | 25.4% | 9.9% |
|  | Selective state school or grammar school | 6.3% | 43.9% | 10.5% | 29.2% | 10.1% |
|  | Private school (fee-paying) | 8.9% | 45.6% | 12.2% | 24.4% | 8.9% |
|  | Prefer not to say | 10.0% | 35.8% | 19.6% | 25.5% | 9.2% |
| *Parent or sibling in Medicine* | |  |  |  |  |  |
|  | Yes | 8.3% | 46.3% | 10.8% | 25.7% | 8.9% |
|  | No | 7.7% | 44.5% | 11.9% | 26.1% | 9.9% |
| *Fee status* | |  |  |  |  |  |
|  | Home | 7.6% | 45.0% | 11.3% | 26.2% | 9.9% |
|  | EU/EEA | 9.6% | 44.4% | 11.3% | 22.9% | 11.9% |
|  | International (non-EU) | 8.9% | 44.0% | 14.8% | 25.8% | 6.4% |
| *Year of study* | |  |  |  |  |  |
|  | Year 1 | 4.5% | 39.3% | 16.7% | 28.3% | 11.3% |
|  | Year 2 | 5.7% | 39.8% | 12.8% | 30.2% | 11.5% |
|  | Year 3 (but not penultimate year) | 5.1% | 40.9% | 11.5% | 30.2% | 12.3% |
|  | Year 4 (but not penultimate or final year) | 8.2% | 41.8% | 13.2% | 27.6% | 9.1% |
|  | Penultimate year | 9.8% | 48.7% | 9.3% | 23.6% | 8.5% |
|  | Final year | 14.0% | 58.1% | 8.0% | 15.6% | 4.3% |
