# Supplementary material for: Specialty choices among UK medical students: certainty, confidence and key influences—a national survey (FAST Study)
Source: BMJ Open. 2025 Aug 8;15(8):e103061. doi: 10.1136/bmjopen-2025-103061 (PMC12336620; doi:10.1136/bmjopen-2025-103061)
Supplement: online supplemental material 8 [file bmjopen-15-8-s008.docx]

| **Characteristic** | | **Very confident** | **Fairly confident** | **Neutral** | **Fairly unconfident** | **Not confident at all** |
| --- | --- | --- | --- | --- | --- | --- |
| *Ethnicity* | |  |  |  |  |  |
|  | Asian or Asian British | 2.8% | 17.3% | 42.8% | 27.1% | 10.0% |
|  | Black, Black British, Caribbean or African | 6.3% | 23.4% | 40.9% | 21.2% | 8.1% |
|  | Mixed or multiple ethnic groups | 3.1% | 20.1% | 41.6% | 23.9% | 11.2% |
|  | White | 3.4% | 21.0% | 38.5% | 28.1% | 9.0% |
|  | Other | 4.5% | 16.1% | 44.1% | 26.0% | 9.3% |
|  | Prefer not to say | 2.1% | 13.4% | 46.4% | 22.7% | 15.5% |
| *Gender* | |  |  |  |  |  |
|  | Female | 2.6% | 18.9% | 39.7% | 28.6% | 10.2% |
|  | Male | 5.0% | 21.7% | 41.9% | 23.8% | 7.6% |
|  | Non-binary | 6.4% | 21.8% | 46.2% | 20.5% | 5.1% |
|  | Prefer not to say | 3.8% | 13.2% | 43.4% | 20.8% | 18.9% |
| *Level of education* | |  |  |  |  |  |
|  | Postgraduate | 5.2% | 23.1% | 36.9% | 24.9% | 9.9% |
|  | Undergraduate | 3.0% | 19.0% | 41.2% | 27.5% | 9.4% |
| *Previous schooling* | |  |  |  |  |  |
|  | Comprehensive state school | 2.8% | 19.2% | 39.3% | 28.0% | 10.6% |
|  | Selective state school or grammar school | 3.6% | 18.8% | 41.2% | 27.7% | 8.8% |
|  | Private school (fee-paying) | 4.2% | 22.0% | 41.1% | 24.7% | 8.0% |
|  | Prefer not to say | 3.7% | 15.9% | 45.8% | 26.6% | 8.1% |
| *Parent or sibling in Medicine* | |  |  |  |  |  |
|  | Yes | 4.4% | 22.7% | 40.8% | 23.7% | 8.3% |
|  | No | 3.1% | 18.9% | 40.3% | 27.9% | 9.7% |
| *Fee status* | |  |  |  |  |  |
|  | Home | 3.1% | 19.8% | 40.1% | 27.5% | 9.6% |
|  | EU/EEA | 4.8% | 19.8% | 36.5% | 30.4% | 8.5% |
|  | International (non-EU) | 5.9% | 19.3% | 44.7% | 21.7% | 8.4% |
| *Year of study* | |  |  |  |  |  |
|  | Year 1 | 3.5% | 16.1% | 50.2% | 21.5% | 8.7% |
|  | Year 2 | 3.4% | 20.1% | 41.2% | 26.0% | 9.2% |
|  | Year 3 (but not penultimate year) | 3.0% | 17.0% | 40.4% | 28.6% | 11.0% |
|  | Year 4 (but not penultimate or final year) | 2.4% | 21.4% | 40.2% | 27.0% | 9.0% |
|  | Penultimate year | 3.5% | 21.2% | 38.1% | 28.5% | 8.7% |
|  | Final year | 4.2% | 23.1% | 33.4% | 29.5% | 9.8% |
