# Supplementary material for: Specialty choices among UK medical students: certainty, confidence and key influences—a national survey (FAST Study)
Source: BMJ Open. 2025 Aug 8;15(8):e103061. doi: 10.1136/bmjopen-2025-103061 (PMC12336620; doi:10.1136/bmjopen-2025-103061)
Supplement: online supplemental material 9 [file bmjopen-15-8-s009.docx]

| **Factors affecting specialty training preference** | | **Influential** | **Neutral** | **Not influential** |
| --- | --- | --- | --- | --- |
| *Work and Training Characteristics* | |  |  |  |
|  | Work-life balance | 79.0% | 12.7% | 8.3% |
|  | Length of specialty training | 44.5% | 28.0% | 27.5% |
|  | Level of stress and pressure at work | 64.7% | 21.8% | 13.5% |
|  | Level of competition for entry into the specialty | 49.8% | 28.9% | 21.3% |
|  | Training structure (run through i.e., entry at ST1 vs. uncoupled i.e., entry via core training and having to reapply at ST3) | 39.6% | 36.9% | 23.5% |
| *Financial Considerations* | |  |  |  |
|  | Financial remuneration | 66.1% | 21.3% | 12.5% |
|  | Potential for private practice earnings | 40.8% | 26.3% | 32.9% |
|  | Number of exams and overall cost of specialty training | 39.4% | 30.5% | 30.1% |
| *Career Security* | |  |  |  |
|  | Future outlook of the specialty | 70.5% | 19.4% | 10.1% |
| *Patient and Clinical Interactions* | |  |  |  |
|  | Continuity of care with patients | 49.2% | 28.0% | 22.8% |
|  | Level of patient interaction | 77.5% | 15.8% | 6.8% |
|  | Diversity of patient interactions | 64.5% | 22.1% | 13.3% |
| *Personal and Lifestyle Considerations* | |  |  |  |
|  | Compatibility with family life | 76.7% | 14.4% | 9.0% |
|  | Out-of-hours demands (OOH shifts) | 61.1% | 24.7% | 14.2% |
|  | Geographic location preference (e.g., tertiary hospital vs district general hospital vs community) | 60.3% | 23.2% | 16.6% |
| *Social and Professional Perception* | |  |  |  |
|  | Perceived prestige of specialty | 20.1% | 23.4% | 56.5% |
|  | Stereotypes surrounding specialty | 15.6% | 24.1% | 60.3% |
| *Gender Split In Specialty* | |  |  |  |
|  | Gender distribution of doctors in the specialty | 21.2% | 28.7% | 50.1% |
| *Demographic Preferences* | |  |  |  |
|  | Preference for working with specific gender groups | 12.4% | 20.5% | 67.1% |
|  | Preference for working with specific age groups (e.g., geriatrics, paediatrics) | 35.3% | 21.5% | 43.2% |
| *Intellectual and Professional Growth* | |  |  |  |
|  | Intellectual challenge | 67.4% | 20.7% | 11.9% |
|  | Research opportunities within specialty | 37.8% | 24.3% | 37.9% |
|  | Use of advanced technology in the specialty | 31.4% | 27.2% | 41.4% |
|  | Use of clinical diagnostic skills vs. investigations | 53.1% | 29.1% | 17.8% |
|  | Interest in specific conditions | 57.1% | 24.7% | 18.2% |
| *Previous Experiences* | |  |  |  |
|  | Personal experiences of disease | 27.0% | 22.8% | 50.1% |
|  | Pre-clinical positive experiences with the specialty (e.g., lectures, tutorials) | 54.9% | 20.9% | 24.1% |
|  | Past positive interactions with the specialty (e.g., rotations, clinical attachments) | 72.0% | 16.5% | 11.5% |
|  | Influence of mentors or role models | 55.0% | 24.2% | 20.8% |
