# Supplementary material for: Specialty choices among UK medical students: certainty, confidence and key influences—a national survey (FAST Study)
Source: BMJ Open. 2025 Aug 8;15(8):e103061. doi: 10.1136/bmjopen-2025-103061 (PMC12336620; doi:10.1136/bmjopen-2025-103061)
Supplement: online supplemental material 10 [file bmjopen-15-8-s010.docx]

| **Factors affecting specialty training preference** | | **Influential** | **Neutral** | **Not influential** |
| --- | --- | --- | --- | --- |
| *Work and Training Characteristics* | |  |  |  |
|  | Work-life balance | 71.1% | 18.1% | 10.7% |
|  | Length of specialty training | 41.7% | 28.9% | 29.5% |
|  | Level of stress and pressure at work | 59.1% | 25.1% | 15.8% |
|  | Level of competition for entry into the specialty | 43.0% | 33.3% | 23.7% |
|  | Training structure (run through i.e., entry at ST1 vs. uncoupled i.e., entry via core training and having to reapply at ST3) | 37.4% | 41.8% | 20.7% |
| *Financial Considerations* | |  |  |  |
|  | Financial remuneration | 63.6% | 21.5% | 14.9% |
|  | Potential for private practice earnings | 40.1% | 29.3% | 30.6% |
|  | Number of exams and overall cost of specialty training | 43.9% | 30.1% | 26.0% |
| *Career Security* | |  |  |  |
|  | Future outlook of the specialty | 69.6% | 19.9% | 10.6% |
| *Patient and Clinical Interactions* | |  |  |  |
|  | Continuity of care with patients | 52.9% | 27.1% | 20.0% |
|  | Level of patient interaction | 75.5% | 17.5% | 7.0% |
|  | Diversity of patient interactions | 62.2% | 23.6% | 14.2% |
| *Personal and Lifestyle Considerations* | |  |  |  |
|  | Compatibility with family life | 71.8% | 18.5% | 9.7% |
|  | Out-of-hours demands (OOH shifts) | 54.6% | 28.9% | 16.5% |
|  | Geographic location preference (e.g., tertiary hospital vs district general hospital vs community) | 56.6% | 24.2% | 19.2% |
| *Social and Professional Perception* | |  |  |  |
|  | Perceived prestige of specialty | 20.4% | 25.7% | 53.9% |
|  | Stereotypes surrounding specialty | 13.1% | 26.2% | 60.7% |
| *Gender Split In Specialty* | |  |  |  |
|  | Gender distribution of doctors in the specialty | 19.2% | 30.4% | 50.5% |
| *Demographic Preferences* | |  |  |  |
|  | Preference for working with specific gender groups | 12.2% | 21.5% | 66.2% |
|  | Preference for working with specific age groups (e.g., geriatrics, paediatrics) | 33.8% | 23.5% | 42.7% |
| *Intellectual and Professional Growth* | |  |  |  |
|  | Intellectual challenge | 67.6% | 21.6% | 10.8% |
|  | Research opportunities within specialty | 41.7% | 26.7% | 31.6% |
|  | Use of advanced technology in the specialty | 41.3% | 27.7% | 30.9% |
|  | Use of clinical diagnostic skills vs. investigations | 55.7% | 30.2% | 14.1% |
|  | Interest in specific conditions | 60.2% | 24.7% | 15.1% |
| *Previous Experiences* | |  |  |  |
|  | Personal experiences of disease | 29.6% | 25.8% | 44.6% |
|  | Pre-clinical positive experiences with the specialty (e.g., lectures, tutorials) | 55.2% | 24.3% | 20.4% |
|  | Past positive interactions with the specialty (e.g., rotations, clinical attachments) | 56.8% | 26.2% | 17.1% |
|  | Influence of mentors or role models | 48.3% | 27.8% | 23.9% |
