# Supplementary material for: Specialty choices among UK medical students: certainty, confidence and key influences—a national survey (FAST Study)
Source: BMJ Open. 2025 Aug 8;15(8):e103061. doi: 10.1136/bmjopen-2025-103061 (PMC12336620; doi:10.1136/bmjopen-2025-103061)
Supplement: online supplemental material 11 [file bmjopen-15-8-s011.docx]

| **Year of Study** | **Very confident** | **Fairly confident** | **Neutral** | **Fairly unconfident** | **Not confident at all** |
| --- | --- | --- | --- | --- | --- |
| Year 1 | 3.5% | 16.3% | 50.0% | 21.3% | 8.8% |
| Year 2 | 3.5% | 20.1% | 40.8% | 26.5% | 9.1% |
| Year 3 (but not penultimate year) | 3.0% | 17.1% | 40.5% | 28.3% | 10.9% |
| Year 4 (but not penultimate or final year) | 2.5% | 22.4% | 38.7% | 27.5% | 9.0% |
| Penultimate year | 3.5% | 21.3% | 38.2% | 28.5% | 8.6% |
| Final year | 4.2% | 23.4% | 33.3% | 29.5% | 9.7% |
