# Supplementary material for: Specialty choices among UK medical students: certainty, confidence and key influences—a national survey (FAST Study)
Source: BMJ Open. 2025 Aug 8;15(8):e103061. doi: 10.1136/bmjopen-2025-103061 (PMC12336620; doi:10.1136/bmjopen-2025-103061)
Supplement: Supplementary file 12 [file bmjopen-2025-103061.reviewer_comments.pdf]

PEER REVIEW HISTORY

BMJ Open publishes all reviews undertaken for accepted manuscripts. Reviewers are asked to complete a checklist review form and are provided with free text boxes to elaborate on their assessment. These free text comments are reproduced below.

ARTICLE DETAILS

Title (Provisional)

Specialty choices among UK medical students: certainty, confidence, and key influences – a national survey (FAST Study)

Authors

Ferreira, Tomas; Collins, Alexander M.; Feng, Oliver; Fortescue, Amelia; French, Benjamin; Handscomb, Arthur; Bolton, Emily; Plumb, Ella; ., the FAST Collaborative

---

VERSION 1 - REVIEW

**Reviewer**	**1**
**Name**	**Eimear Spain**
**Affiliation**	**University of Limerick**
**Date**	**16-Jan-2025**
**COI**	**None**

General Comments
An interesting article with a strong data set. Given current workforce issues it makes a timely contribution to knowledge, which will no doubt be of interest to readers. However, the results and discussion sections need some work. Of particular importance is greater engagement with and linking of the existing literature with results in the discussion section. What does the data tell us about what is going on? Further detail below.

Introduction
• Set the scene before detailing training routes.
• Reference is made to this being "this is the largest ever study of UK medical students' specialty preferences…" and later "To our knowledge, this is…" remove the latter.

Results
• References for 'sister publications' to be inserted before publication.
• P13 36/37 typo, reference to which previously published paper?
• P13 What do you mean by "geographic location preferences"
• "Compatibility with family life was significantly influential, with 37.3% finding it 'fairly influential' and 39.4% 'very influential'. The continuity of care with patients and out-of-hours demands were considered influential by 49.3% and 61.2%, respectively. Geographic location preferences were important to 60.3% of students." Preceding sentence relates to gender differences – are these female only? Clarify.
• P14 discussion could be clearer. Review. Shorter paragraphs, discuss thematically (perhaps reflective of themes identified in table 3?).
• P18 review figure numbering/headings

Discussion
• P 20 typo line 27,28
• P20 "However, despite increasing popularity among specialty trainees of all genders and backgrounds, stigma persists (particularly in surgical specialties) which serves as a barrier to its uptake." Popularity of what?
• P21 "Factors" what does this refer to?
• Separate discussion/heading for gender differences?
• Greater engagement with the literature in the discussion section- what does the data tell us about what is going on? See above
• Change heading to "Speciality specific factors" rather than Radar Plots?

Recommendations
Recommendations should be expanded to reflect earlier discussion, for example, role of medical schools, identification of bottlenecks etc.

**Reviewer**	**2**
**Name**	**Charles Weissman**
**Affiliation**	**Hebrew University-Hadassah School of Medicine**
**Date**	**16-Jan-2025**
**COI**	**None**

Comments to the Author:
This manuscript is the report of a survey performed among British medical students concerning the process and criteria of medical specialty selection. It is 19 single spaced pages long with little focus and cohesion. It reads like a compilation of many observations and ideas without an overall practical conclusion. What is the reader to learn from this manuscript?
A. The manuscript is too long with a tremendous amount of data presented. Therefore, more of the results should be placed in tables and figures. Only the results that lead to conclusions and recommendations should be presented. The amount of data presented is out of proportion to the concrete conclusions and recommendations. The discussion should be shortened. The aim should be not to present all the data collected, but to focus just on the data that is most important to reaching clear conclusions.
B. "Additionally, readers may find further insights in our sister publication, which examines the role of extracurricular achievements in shaping medical students' career trajectories" The authors wrote, but not yet published, another manuscript using the same data. This manuscript should be provided to the reviewers to determine if it is a duplicate publication.
C. The results of this survey essentially confirm the results of previous smaller surveys. The authors need to better show what is unique and new about the results of this study. For example, it has been repeatedly shown that a controllable lifestyle is a very important specialty selection criterion, which this study confirms. Is there anything in this study which adds or is different than the results of previous research?
D. The recommendations to the healthcare and medical education leaderships are confined to a paragraph advocating more part-time residency positions, while some other recommendations are buried in the Discussion and Conclusions. The recommendations should be better presented and justified. They should also include ideas of how to implement them.
E. It is important to point out the nature of the methodology: A cross-sectional convenience sample obtained via the Internet. Therefore, there was no control over which students answered and how many. This should be discussed.
Specific Comments
Introduction: "However, there is a notable lack of high-powered studies that comprehensively summarise the specialty preferences of UK medical students across all years of study and explore the factors underpinning these decisions" – define "high-powered" – do you mean highly powered from the

point of sample size or high-powered in another way?
Introduction: "As alluded to above, an improved understanding of students' intended career paths would be of value to various stakeholders in healthcare and medical education. For medical schools…" – This paragraph discusses a variety of possible policy and procedural implications of this study in attempt to justify its importance. These ideas and recommendations should be placed in the Discussion Section and better explained.
Methods: "All participants were asked about their involvement in various extracurricular activities which at the time of survey design, were measures used in candidate selection for specialist training." Is this relevant to the present study? Is it not the subject of the other manuscript by the same authors? If it is not relevant then the entire section on extracurricular activities should be eliminated.
Discussion: "Notably, a very low proportion of students across all year groups expressed confidence in their ability to secure a specialty training place, likely influenced by current training bottlenecks and competition ratios. The study highlights the need for improved career guidance and broader exposure to specialties throughout medical school." How does this study highlight the need for better career guidance when the bottlenecks and insufficient training spaces are present? Please explain. Also, how will more exposure to specialties affect the selection, to which specialties is there insufficient exposure?
Discussion: "increased guidance, insight, and networking" - a recurrent theme is the need for increase guidance. Please give details about who should provide the guidance, when should it be provided an what should be the content of the guidance.

**Reviewer**	**3**
**Name**	**Kyle Dack**
**Affiliation**	**University of Bristol**
**Date**	**16-Jan-2025**
**COI**	**None**

Comments to the Author:
The manuscript bmjopen-2024-093789 is related to bmjopen-2024-093196, and the editorial team asked me to comment on the overlap between the two manuscripts and whether they are appropriate as separate publications. The data source, predictor variables, and the statistical methods are the same in both papers, and much of the methods/results sections are identical. Therefore I think a much higher impact paper could be made by merging the two manuscripts.

However, this is precluded by neither paper being suitable for publication due to serious issues with the statistical methodology. Namely (a) incomplete methods section (please refer to STROBE guidelines for details) (b) the lack of a theoretical justification for the model covariates (stepwise selection) and (c) a high risk of confounding bias.

Abstract
1. What is described as "outcomes" are in fact the aims of the study. The outcome is specialty choice.

2. Some findings are reported with statistics, but some are not.

Strengths and weaknesses section
3. "Reliance on self-reported data may introduce some bias, but the large sample size and anonymity likely mitigated these effects" I disagree with this. Bias due to self reporting can be present regardless of sample size. I am not sure how anonymity would alleviate this.

Introduction
4. Aims are described as outcomes – an outcome is a measured variable.

5. There is a lack of explanation for what factors the study wishes to evaluate, and what prior research tells us about them.

6. Abbreviations should be explained at first use.

7. The final reference is missing.

Methods
8. There are important sections missing concerning the details of the outcomes and predictors, which are required by the STROBE checklist. These are marked as completed in STROBE but this is not true, there is no information about how the outcome was defined measured or categorised, and what predictor variables were selected.

9. The data analysis section is incomplete, with much of the details of the study missing. Every analysis should be reported with enough detail for others to evaluate or replicate the analysis. After reading the introduction and methods the reader will still not know what is being modelled.

10. There is a lack of consideration for confounding bias. What factors could influence both the predictor and outcome? Additionally, it is not described how multivariable models were constructed but I assume it is the same stepwise method as in the other paper, which is not suitable for inference.

Results
11. Much of my feedback is the same as in the other paper as the results are identical.

12. Decimal places are not consistent between statistics.

13. Various odds ratios are reported but there is no information provided about what the models are, including which covariates, and using what statistical methods.

Discussion
14. There is not enough information about the statistical methods for me to evaluate whether the interpretation is valid or not.

VERSION 1 - AUTHOR RESPONSE

Reviewer 1:

Comment 1: Set the scene before detailing training routes.

> Response: We agree that the introduction would benefit from additional context. We have expanded on the broader importance of specialty decision-making in the UK medical training system and the current challenges within the postgraduate training landscape. This provides clearer justification for our study prior to detailing specific training routes.

Comment 2: Reference is made to this being “this is the largest ever study of UK medical students' specialty preferences…” and later “To our knowledge, this is…” remove the latter.

> Response: We have removed the phrase “To our knowledge, this is…” to avoid redundancy and maintain clarity.

Comment 3: References for ‘sister publications’ to be inserted before publication.

Response: We confirm that references to our sister publications will be inserted prior to final submission, once citation details (including DOI) are confirmed.

Comment 4: P13 36/37 typo, reference to which previously published paper?

Response: We have corrected the typographical error and clarified the reference to the previously published paper.

Comment 5: P13 What do you mean by “geographic location preferences”?

Response: We now clarify in the results section that “geographic location preferences” refers to students’ preferences for training or practising in particular types of settings (e.g. urban vs rural) or specific regions of the UK.

Comment 6: “Compatibility with family life was significantly influential, with 37.3% finding it 'fairly influential' and 39.4% 'very influential'. The continuity of care with patients and out-of-hours demands were considered influential by 49.3% and 61.2%, respectively. Geographic location preferences were important to 60.3% of students.” Preceding sentence relates to gender differences – are these female only? Clarify.

Response: Thank you for noting this. We have clarified in the text that the percentages cited reflect responses from the whole cohort. Where gender-specific findings are presented, this is now explicitly stated.

Comment 7: P14 discussion could be clearer. Review. Shorter paragraphs, discuss thematically (perhaps reflective of themes identified in Table 3?).

Response: We have revised the discussion section for improved clarity and flow. Paragraphs have been shortened and reorganised thematically, drawing on the structure of Table 3. This helps to better synthesise key patterns and influences identified in the data.

Comment 8: P18 review figure numbering/headings.

Response: We have reviewed all figure numbering and headings for accuracy and consistency throughout the manuscript.

Comment 9: P20 typo line 27,28.

Response: The typographical error has been corrected in the revised manuscript.

Comment 10: P20 “However, despite increasing popularity among specialty trainees of all genders and backgrounds, stigma persists (particularly in surgical specialties) which serves as a barrier to its uptake.” Popularity of what?

Response: We have amended the sentence to clarify that it refers to the increasing popularity of Less Than Full-Time (LTFT) training among trainees of all genders and backgrounds.

Comment 11: P21 “Factors” – what does this refer to?

Response: We have specified the factors referred to on Page 21.

Comment 12: Separate discussion/heading for gender differences?

Response: We agree that the gender-related findings merit dedicated discussion. A new subheading has been added within the discussion section to highlight and explore these results more clearly.

Comment 13: Greater engagement with the literature in the discussion section – what does the data tell us about what is going on? See above.

Response: We have expanded the discussion to incorporate a deeper engagement with existing literature. We draw comparisons with previous studies and offer potential explanations for our findings, supported by current policy discussions and workforce reports.

Comment 14: Change heading to "Specialty-specific factors" rather than Radar Plots?

Response: We have changed the heading from "Radar Plots" to better align with the content and improve readability.

Comment 15: Recommendations should be expanded to reflect earlier discussion, for example, role of medical schools, identification of bottlenecks etc.

Response: We have expanded and consolidated the recommendations into a clearer section. This now includes the role of medical schools, professional institutions, the need for earlier and more structured career guidance, and the importance of addressing perceived and actual bottlenecks in the specialty training pipeline. Suggested implementation strategies are also included.

Reviewer 2:

Comment 1: The manuscript is too long with a tremendous amount of data presented. Therefore, more of the results should be placed in tables and figures. Only the results that lead to conclusions and recommendations should be presented. The amount of data presented is out of proportion to the concrete conclusions and recommendations. The discussion should be shortened. The aim should be not to present all the data collected, but to focus just on the data that is most important to reaching clear conclusions.

Response: We agree that a more concise presentation will strengthen the manuscript. We have revised the results section to prioritise findings that directly support our key conclusions and recommendations. Where possible, additional data has been relocated to supplementary tables or figures. The discussion section has also been shortened and refocused to align more clearly with the core themes emerging from the results.

Comment 2. "Additionally, readers may find further insights in our sister publication, which examines the role of extracurricular achievements in shaping medical students' career trajectories" The authors wrote, but not yet published, another manuscript using the same data. This manuscript should be provided to the reviewers to determine if it is a duplicate publication.

Response: This was out of our control and an editor choice.

Comment 3: The results of this survey essentially confirm the results of previous smaller surveys. The authors need to better show what is unique and new about the results of this study. For example, it has been repeatedly shown that a controllable lifestyle is a very important specialty selection criterion, which this study confirms. Is there anything in this study which adds or is different than the results of previous research?

Response: We have revised the discussion section to more clearly highlight the novel contributions of this study, including: (1) the size and diversity of the sample, (2) inclusion of students across all years of study, and (3) new insights into the influence of geography, family background, and career confidence on specialty preference. These findings are explicitly compared with existing literature to underscore what is distinct or additive.

Comment 4. The recommendations to the healthcare and medical education leadership are confined to a paragraph advocating more part-time residency positions, while some other recommendations are buried in the Discussion and Conclusions. The recommendations should be better presented and justified. They should also include ideas of how to implement them.

Response: We have consolidated all recommendations into a new, clearly labelled section. This now includes a rationale for each recommendation, as well as suggested strategies for implementation—such as improved access to LTFT training, structured specialty exposure programmes in earlier years of medical school, and targeted career guidance for underrepresented groups.

Comment 5. It is important to point out the nature of the methodology: A cross-sectional convenience sample obtained via the Internet. Therefore, there was no control over which students answered and how many. This should be discussed.

Response: We agree and have expanded the limitations section to clarify the implications of using a cross-sectional convenience sample. Specifically, we note the inability to determine a precise response rate or ensure representativeness. Nonetheless, we highlight that the sample is large, geographically diverse, and includes students across all years of study, which mitigates some concerns.

Comment 6: Introduction: "However, there is a notable lack of high-powered studies that comprehensively summarise the specialty preferences of UK medical students across all years of study and explore the factors underpinning these decisions" – define "high-powered" – do you mean highly powered from the point of sample size or high-powered in another way?

Response: We have revised the sentence to avoid ambiguity. We now state that there is a lack of "large-scale, statistically well-powered studies" to clarify that the reference is to sample size and statistical robustness.

Comment 7: Introduction: "As alluded to above, an improved understanding of students' intended career paths would be of value to various stakeholders in healthcare and medical education. For medical schools…" – This paragraph discusses a variety of possible policy and procedural implications of this study in attempt to justify its importance. These ideas and recommendations should be placed in the Discussion Section and better explained.

Response: We have removed the policy recommendations from the Introduction and repositioned them in the Discussion, where they are now developed in more depth and aligned with our key findings.

Comment 8: Methods: "All participants were asked about their involvement in various extracurricular activities which at the time of survey design, were measures used in candidate selection for specialist training." Is this relevant to the present study? Is it not the subject of the other manuscript by the same authors? If it is not relevant then the entire section on extracurricular activities should be eliminated.

Response: We acknowledge that this content is more directly relevant to the sister manuscript and as such we have removed it.

Comment 9: Discussion: "Notably, a very low proportion of students across all year groups expressed confidence in their ability to secure a specialty training place, likely influenced by current training bottlenecks and competition ratios. The study highlights the need for improved career guidance and broader exposure to specialties throughout medical school." How does this study highlight the need for better career guidance when the bottlenecks and insufficient training spaces are present? Please explain. Also, how will more exposure to specialties affect the selection, to which specialties is there insufficient exposure?

Response: We have clarified and streamlined our rationale.

Comment 10: Discussion: "increased guidance, insight, and networking" - a recurrent theme is the need for increase guidance. Please give details about who should provide the guidance, when should it be provided an what should be the content of the guidance.

Response: We have revised these sections to include clearer recommendations. Specifically, we propose that structured career guidance should be led by medical schools from early undergraduate years, incorporating input from clinicians, trainees, and specialty-specific societies. Key content areas include training pathways, competition ratios, lifestyle implications, and workforce needs.

Reviewer 3:

Comment 1: The data source, predictor variables, and the statistical methods are the same in both papers, and much of the methods/results sections are identical. Therefore I think a much higher impact paper could be made by merging the two manuscripts.

Response: We acknowledge that the data source and some variables are shared between the two manuscripts. However, the research aims, analytical approaches, and outcomes assessed differ significantly. This manuscript focuses on specialty training preferences, confidence and certainty, while the other investigates the influence of extracurricular achievements on career trajectories. Both papers provide distinct and valuable insights. We have removed overlapping results.

Comment 2: However, this is precluded by neither paper being suitable for publication due to serious issues with the statistical methodology. Namely (a) incomplete methods section (please refer to STROBE guidelines for details) (b) the lack of a theoretical justification for the model covariates (stepwise selection) and (c) a high risk of confounding bias.

Response:

(a) We have revised the methods section to fully comply with the STROBE checklist. Specifically, we have added detailed descriptions of the outcomes, predictor variables and statistical analyses to the Methods and Data Analysis sections.

(b) We have carefully justified our choice of covariates based on prior research and knowledge of the medical profession. All of our multivariable logistic models include the same set of demographic and educational predictors. We no longer use stepwise regression or AIC to address the reviewer's valid concerns about post-selection inference among other things, although more generally we maintain that data-driven model selection is informative and often essential in statistics and machine learning if applied with care.

(c) In our informed judgement, the main sources of confounding bias have been accounted for, but we discuss this point in more detail in the Limitations section, In this

paper, we refrain from making definite causal statements, and instead interpret odds ratios within the context of the logistic regression model whose assumptions and limitations we have explained at length. A more refined analysis would posit or learn an underlying causal structure (direct acyclic graph) and estimate direct and indirect effects (including through mediators) in a more transparent way, but we do not pursue this here.

Comment 3: What is described as "outcomes" are in fact the aims of the study. The outcome is specialty choice.

Response: Thank you for this clarification. We have revised to accurately refer to "specialty choice" as the primary outcome, and have ensured the study aims are separately and clearly stated.

Comment 4:. Some findings are reported with statistics, but some are not.

Response: We have reviewed to ensure consistency.

Comment 5. "Reliance on self-reported data may introduce some bias, but the large sample size and anonymity likely mitigated these effects" I disagree with this. Bias due to self reporting can be present regardless of sample size. I am not sure how anonymity would alleviate this.

Response: We agree that self-reporting bias cannot be fully mitigated by sample size or anonymity. We have revised the relevant sentence in the Limitations section to state that: "While anonymity may reduce social desirability bias, self-reported data remain subject to recall error and subjective interpretation. These are inherent limitations of survey-based research."

Comment 6. Aims are described as outcomes – an outcome is a measured variable.

Response: We have revised the introduction to clearly distinguish between the aims of the study and the measured outcome (specialty choice).

Comment 7: There is a lack of explanation for what factors the study wishes to evaluate, and what prior research tells us about them.

Response: We have expanded the introduction and methods to specify the factors being evaluated (e.g., gender, lifestyle preferences, geographic location) and summarised prior research to contextualise these variables. Additionally, readers are signposted to our protocol.

Comment 8. Abbreviations should be explained at first use.

Response: We have reviewed the manuscript to ensure all abbreviations (e.g., LTFT, FY1, ST) are clearly defined upon first use.

Comment 9: The final reference is missing.

Response: Addressed.

Comment 10: There are important sections missing concerning the details of the outcomes and predictors, which are required by the STROBE checklist. These are marked as completed in STROBE but this is not true, there is no information about how the outcome was defined, measured or categorised, and what predictor variables were selected.

Response: We have undertaken a thorough revision to ensure full compliance with the STROBE checklist. In particular, the methods section has now been expanded to include detailed definitions of the various outcomes and justify our choice of predictor variables. We explain in the Data Analysis section how we transform the 5-point ordinal responses into a binary variable to which we apply logistic regression.

Comment 11. The data analysis section is incomplete, with much of the details of the study missing. Every analysis should be reported with enough detail for others to evaluate or replicate the analysis. After reading the introduction and methods the reader will still not know what is being modelled.

Response: The Data Analysis section has been extensively revised. It now details the use of multivariable logistic regression to model binary outcomes (e.g., high vs. low influence of particular factors on specialty choice), including the covariates included in all models. We clarify that all models adjust for a consistent set of predictors and explicitly describe our variable transformations. Full model outputs are included in the supplementary materials.

Comment 12. There is a lack of consideration for confounding bias. What factors could influence both the predictor and outcome? Additionally, it is not described how multivariable models were constructed but I assume it is the same stepwise method as in the other paper, which is not suitable for inference.

Response: We have expanded our discussion of potential confounders in the limitations section. As mentioned above, we control for all other demographic and educational predictors when making comparisons between e.g. male and female students and emphasise in the text that the estimated odds ratios should be interpreted in this context.

Comment 13. Much of my feedback is the same as in the other paper as the results are identical.

Response: We acknowledge that the datasets overlap but emphasise that the results focus on different research questions which is evident. We have ensured clear distinctions are made in the results sections of both manuscripts.

Comment 14. Decimal places are not consistent between statistics.

Response: This has been standardised. As stated in the revised Data Analysis section, percentages are now rounded to one decimal place, and odds ratios and 95% confidence intervals are reported to two decimal places throughout.

Comment 15. Various odds ratios are reported but there is no information provided about what the models are, including which covariates, and using what statistical methods.

Response: We have addressed this by explicitly outlining the structure of the multivariable logistic regression models in the Methods and Data Analysis sections. For each outcome, we describe the predictors included and we included the entire html of the analysis in the supplementary material.

Comment 16. There is not enough information about the statistical methods for me to evaluate whether the interpretation is valid or not.

Response: We have expanded the Data Analysis and Discussion sections to provide clearer, more transparent justification of our analytical approach. We also explicitly link the results presented to the models used, ensuring readers can confidently assess the appropriateness and limitations of our interpretations.

VERSION 2 - REVIEW

Reviewer 2
Name Weissman , Charles
Affiliation Hebrew University-Hadassah School of Medicine, Anesthesiology and Critical Care Medicine
Date 23-May-2025
COI

The reviewers' comments have been addressed satisfactorily.

Reviewer 3
Name Dack, Kyle
Affiliation University of Bristol, MRC Integrative Epidemiology Unit
Date 11-May-2025
COI

Thank you to the authors for making extensive revisions, this significantly strengthens the reliability of the findings and makes the methodology more transparent.

Given the length of time since the first review and the amount of changes, I have performed a full second review. I have only a few minor comments:

1. Figure 2. This is a very nice way of showing the results, and it looks interesting, but what do the numbers represent? I suggest adding a lay explanation to help readers interpret this, where figure 2 is referenced. I think this should also be part of the statistical analysis section of the methods. The text in the final two plots is a different colour to the others.

2. “Additionally, confidence was notably higher among those who had completed an intercalated or previous degree (OR 1.22, CI [1.08, 1.37], p<0.0001) and those with a PubMed-indexed publication (OR 1.67, CI [1.393, 2.00], p<0.0001).” The second LCI should be rounded to 1.39 for consistency.

3. Page 14 line 60, page 15 line 17: the confidence intervals contain a zero which has been cut off, e.g. “3.00” became “3”. The zeroes should be added to ensure readability.

4. Discussion: the radar plots are discussed in the subheading “Specialty-specific influencing factors” but the plot is referred to as “these plots”. This is quite a long way into the discussion and readers may not know what plots are being referred to – suggest clarifying.

VERSION 2 - AUTHOR RESPONSE

Reviewer 2 – Prof. Charles Weissman

“The reviewers' comments have been addressed satisfactorily.”

Response:

We thank Reviewer 2.

Reviewer 3– Dr. Kyle Dack

Comment 1:

“Figure 2. This is a very nice way of showing the results, and it looks interesting, but what do the numbers represent? I suggest adding a lay explanation to help readers interpret this, where figure 2 is referenced. I think this should also be part of the statistical analysis section of the methods. The text in the final two plots is a different colour to the others.”

Response:

Thank you for this helpful suggestion. We have:

- Added a lay explanation of Figure 2 in the Results section, clarifying that the numbers represent average domain scores based on Likert-scale ratings.

- Added a dedicated paragraph under the “Data Analysis” section of the Methods titled “Radar plot generation”, explaining how the composite scores were calculated and plotted.

- Standardised the text colour across all radar plots to ensure consistency, correcting the discrepancy in the final two panels.

Comment 2:

“Additionally, confidence was notably higher among those who had completed an intercalated or previous degree (OR 1.22, CI [1.08, 1.37], p<0.0001) and those with a PubMed-indexed publication (OR 1.67, CI [1.393, 2.00], p<0.0001).” The second LCI should be rounded to 1.39 for consistency.”

Response:

Amended as suggested. Thank you.

Comment 3:

“Page 14 line 60, page 15 line 17: the confidence intervals contain a zero which has been cut off, e.g. “3.00” became “3”. The zeroes should be added to ensure readability.”

Response:

We have carefully reviewed the manuscript and corrected this.

Comment 4:

“Discussion: the radar plots are discussed in the subheading “Specialty-specific influencing factors” but the plot is referred to as “these plots”. This is quite a long way into the discussion and readers may not know what plots are being referred to – suggest clarifying.”

Response:

We have revised the sentence to explicitly refer back to Figure 2, so that readers clearly understand which visualisation is being discussed.
